# Knockdown of IGF2BP2 overcomes cisplatin-resistance in lung cancer through downregulating Spon2 gene

**DOI:** 10.1186/s41065-024-00360-w

**Published:** 2024-12-28

**Authors:** Shilei Zhang, Ting Dou, Hong Li, Hongfang Yu, Wei Zhang, Liping Sun, Jingwen Yang, Zhenfei Wang, Hao Yang

**Affiliations:** 1 Department of Radiation Oncology, Peking University Cancer Hospital (Inner Mongolia Campus) & Affiliated Cancer Hospital of Inner Mongolia Medical University, Inner Mongolia Autonomous Region, Hohhot, 010020 China; 2 Key Laboratoy of Radiation Physics and Biology of Inner, Mongolia Medical University, Peking University Cancer Hospital (Inner Mongolia Campus) & Affiliated Cancer Hospital of Inner Mongolia Medical University, Inner Mongolia Autonomous Region, Hohhot, 010020 China; 3 Department of Oncology, Xilingol League Central Hospital, Xilingol, 026000 China; 4 Department of Graduate School, Inner Mongolia Medical University, Hohhot, Inner Mongolia Autonomous Region 010020 China; 5 The Laboratory for Tumor Molecular Diagnosis, Peking University Cancer Hospital (Inner Mongolia Campus) & Affiliated Cancer Hospital of Inner Mongolia Medical University, Inner Mongolia Autonomous Region, Hohhot, 010020 China; 6 Department of Radiation Oncology, Peking University Cancer Hospital (Inner Mongolia Campus) & Affiliated Cancer Hospital of Inner Mongolia Medical University, Inner Mongolia Autonomous Region, No. 42, Zhaowuda Road, Saihan District, Hohhot, 010000 China

**Keywords:** Lung cancer, N6-methyladosine RNA methylation, Cisplatin, Resistance, IGF2BP2

## Abstract

**Background:**

Cisplatin (DDP) resistance has long posed a challenge in the clinical treatment of lung cancer (LC). Insulin-like growth factor 2 binding protein 2 (IGF2BP2) has been identified as an oncogenic factor in LC, whereas its specific role in DDP resistance in LC remains unclear.

**Results:**

In this study, we investigated the role of IGF2BP2 on DDP resistance in DDP-resistant A549 cells (A549/DDP) in vitro and in a DDP-resistant lung tumor-bearing mouse model in vivo. Additionally, methylated RNA immunoprecipitation sequencing (MeRIP-seq) was conducted to identify the potential mRNAs regulated by IGF2BP2, an N6-methyladenosine (m6A) regulator, in the tumor tissues of mice. Compared to normal tissues, IGF2BP2 levels were increased in LC tissues and in relapsed/resistant LC tissues. Most importantly, IGF2BP2 levels were significantly higher in relapsed/resistant LC tissues than in LC tissues. Significantly, knockdown of IGF2BP2 or DDP treatment inhibited A549 cell viability, migration, and cell cycle progression. Consistently, DDP treatment suppressed the viability and migration and triggered cell cycle arrest in A549/DDP cells in vitro, as well as reduced tumor volume and weight of A549/DDP tumor-bearing mice; meanwhile, the combination of DDP and IGF2BP2 siRNA further significantly inhibited A549/DDP cell growth in vitro and in vivo compared to DDP treatment alone. Furthermore, MeRIP-seq data showed that IGF2BP2 downregulation remarkably elevated m6A levels of spondin 2 (Spon2) and reduced mRNA levels of Spon2 in tumor tissues from A549 tumor-bearing mice. Meanwhile, the combination of DDP and IGF2BP2 siRNA notably reduced Spon2 levels, as well as inhibited the viability and induced apoptosis in A549/DDP cells; however, these effects were reversed by Spon2 overexpression.

**Conclusion:**

Collectively, downregulation of IGF2BP2 could overcome DDP resistance in LC through declining the Spon2 gene expression in an m6A-dependent manner. These results may provide a new strategy for overcoming DDP resistance in LC.

**Supplementary Information:**

The online version contains supplementary material available at 10.1186/s41065-024-00360-w.

## Background

Lung cancer (LC) is the most common malignancy and the primary cause of cancer-related mortality globally [1, 2]. LC is categorized into two main categories: small cell lung cancer (SCLC) and non-small-cell lung carcinoma (NSCLC) [3]. SCLC accounts for approximately 13–15% of all LC cases [1, 4, 5], whereas NSCLC onstitutes about 85% of cases [3]. LC is characterized by a poor prognosis and a propensity to metastasize to distant sites [6, 7]. The five-year survival rate for patients with LC is only about 10% [8, 9]. Although advanced therapeutic options such as radiation therapy, immunotherapy and targeted therapy are available [10], chemotherapy is indispensable for LC treatment [11].

Cisplatin (DDP) has been approved as an anti-tumor drug for the treatment of LC for nearly half a century [12]. DDP binds to nuclear DNA, inducing cell cycle arrest and apoptosis, thus exhibiting its anti-tumor properties [13]. Nevertheless, the development of acquired drug resistance has significantly limited the effectiveness of DDP in clinical cases [14]. Recently, various strategies have been proposed to overcome DDP resistance in LC [15, 16]. For instance, the combination of metformin with DDP has been shown to exert enhanced anti-tumor effects in cancer cells compared to DDP treatment alone [16]. Moreover, aspirin, a non-steroidal anti-inflammatory drug, has been reported to increase the sensitivity of lung cancer cells to DDP [15]. Furthermore, propofol, an anesthetic, has demonstrated the ability overcome DDP resistance in LC [17]. Despite these advancements, the outcomes for LC patients remain unsatisfactory, highlighting the need for further countermeasures.

Epigenetic dysregulation, including RNA modification, plays a crucial role in tumor resistance to anti-cancer therapies [18, 19]. *N*^6^-methyladenosine (m6A) is a specific type of internal mRNA modification [20]. This modification can modulate the translocation, degradation, stability and translation of mRNA, thereby impacting gene expression [21, 22]. The modification of m6A is regulated by demethylases (erasers), methyltransferases (writers), and RNA reader proteins (readers) [23]. Insulin-like growth factor 2 binding protein 2 (IGF2BP2) functions as a specific m6A “reader” [24], and has been implicated in LC progression [25, 26]. Studies have shown that IGF2BP2 can promote the growth of NSCLC cells [27], and is associated with drug resistance in various human cancers [28]. For instance, Han et al. found that forced expression of IGF2BP2 could enhance chemoresistance in glioma cells via stabilizing the long non-coding RNA DANCR [28]. Additionally, IGF2BP2 overexpression has been shown to increase resistance to DDP in cervical cancer cells [29]. However, it remains unclear whether IGF2BP2 influences DDP resistance in LC cells. Therefore, we investigated the role of IGF2BP2 in DDP resistance in LC.

## Materials and methods

### Cell culture and transfection

Human SCLC cell lines H446 (No. CL-0401, Procell), SBC-2 (a kind gift from Tianjin International Joint Academy of Biomedicine), and NSCLC cell lines HCC827 (No. CL-0094, Procell), A549 (No. CL-0016, Procell), H322 (No. 95111734, European Collection of Authenticated Cell Cultures), NCI-H3255 (No. IM-H122, IMMOCELL) and DDP-resistant A549 cells (A549/DDP, No. CL-0519, Procell), human bronchial epithelial cell line (BEAS-2B, No. GDC0139, China Center for Type Culture Collection) were cultured in Dulbecco’s modified Eagle medium/Nutrient Mixture F-12 (DMEM/F-12, C11330500BT, Gibco) supplemented with 10% fetal bovine serum (FBS, No. 10270-106, Gibco) and 1% penicillin/streptomycin, and incubated in an incubator at 37 °C with 5% CO_2_. All cell lines were authenticated by short tandem repeat (STR) profiling.

Small interfering RNAs specifically targeting IGF2BP2 (si-IGF2BP2), siRNA negative control (si-NC), and pcDNA3.1-NC (oe-NC), pcDNA3.1-IGF2BP2 (oe-IGF2BP2) and pcDNA3.1-Spon2 (oe-Spon2) were obtained from HanBio, and then transfected into cells. The sequences of si-IGF2BP2, oe-IGF2BP2, oe-Spon2 have been provided in Table S1.

The si-IGF2BP2 sequences or si-NC sequences were separately cloned into the PGMLV-6751 lentiviral vector (Lv-si-IGF2BP2 or Lv-si-NC). HEK293T cells were transfected with Lv-si-IGF2BP2 or Lv-si-NC plasmids using Lipofectamine2000 for 72 h. Following this, virus particles were harvested, and transduced into A549 or A549/DDP cells for an additional 72 h. Cells were then selected with puromycin for two weeks to obtain stable cell lines.

### Reverse transcription-quantitative polymerase chain reaction (RT-qPCR)

Total RNA was extracted using the Redzol reagent (No. FTR-50, SBS Genetech Co., Ltd.). Next, the first-strand cDNA was synthesized using the Surescript™ First-Strand cDNA Synthesis Kit (No. QP057, iGeneBio). The SYBR Green qPCR Master Mix (None ROX) kit (No. G3320-05, Servicebio) was then utilized for qPCR analysis on an iQ5 Real-Time PCR system (Applied Biosystems). GAPDH served as the internal control for IGF2BP2. The relative IGF2BP2 mRNA level was calculated by the 2^−ΔΔCT^ method, as described by Livak and Schmittgen [30]. The primers used were as follows: IGF2BP2: forward, 5’-GGGACAGTGGAGAATGTGGA − 3’and reverse, 5’-AACTGATGCCCGCTTAGCTT-3’; Spon2: forward, 5’-CTACTGTATGCCAGCCGTGG-3’ and reverse, 5’-CCGCCTCGATCTCCTTCATC-3’; GAPDH: forward, 5’-TCAGCAATGCCTCCTGCAC-3’and reverse, 5’-TCTGGGTGGCAGTGATGGC − 3’.

### Western blot assay

Protein samples were extracted using the Radio-Immunoprecipitation Assay (RIPA) buffer (No. P0013B, Beyotime) and quantified using a Bradford protein concentration assay kit (No. AR0145, BOSTER). Next, the protein samples were separated by 10% sodium dodecyl sulfate-polyacrylamide gel electrophoresis (SDS-PAGE) and then transferred onto polyvinylidene fluoride (PVDF) membranes. The membranes were then incubated with primary antibodies, followed by incubation with the corresponding secondary antibody (No. ab7090, Abcam). Signal enhancement was achieved using an Western Lightning™ Chemiluminescence Reagent (PerkinElmer), and imaging was performed using an Epson Perfection V39 scanner (EPSON). The antibodies utilized in this study included anti-IGF2BP2 (No. ab124930, Abcam), anti-Spon2 (ab171955, Abcam), anti-β-actin (No. ab8227, Abcam) and anti-GAPDH (No. ab9485, Abcam) antibodies.

### 3-(4,5-dimethylthiazol-2-yl)-2,5-diphenyltetrazolium bromide (MTT) assay

A549 and A549/DDP cells were plated onto 96-well plates and incubated overnight. Following this, the cells were cultured for 24, 48 and 72 h. Next, 10 µl of the MTT reagent (No. AR1156, BOSTER) was added to each well and incubated for an additional 4 h. After removing the medium from each well, 100 µL of dimethyl sulfoxide (DMSO) was added. The plate was then placed on a shaker for 10 min to dissolve the formazan crystals. Subsequently, a microplate reader (DNM-9602; PERLONG) was used to measure the optical density (OD) at 570 nm.

### Flow cytometry assay

For the analysis of cell cycle distribution, a Cell cycle staining Kit (No. CCS012, MULYI SCIENCES) was used. A549 and A549/DDP cells were re-suspended in pre-cold phosphate buffered saline (PBS) and fixed in anhydrous ethanol at 4 °C overnight. Subsequently, 50 µg/ml of propidium iodide (PI, provided by MULYI SCIENCES) and 100 µg/ml of RNase A were added into the cell suspension, and the mixture was then incubated for 30 min at 4 °C in the dark. For detecting cell apoptosis, an Annexin V-FITC/PI apoptosis kit (No. AP101-100-kit, MULYI SCIENCES) was utilized. Briefly, cells were stained with 5 µL of Annexin V-FITC and 10 µL of PI for 20 min in a dark environment. Finally, the distribution of cell cycle and cell apoptosis was analyzed using a BD FACSCalibur™ Flow Cytometer (E97501093, BD Biosciences).

### Wound healing assay

A549 and A549/DDP cells were plated onto 12-well plates and allowed to adhere overnight. A sterilized pipette tip was used to create a scratch in the center of each well. Next, cells were washed three times with PBS to remove the suspended cells, after which serum-free medium was added. After incubation of 0 and 24 h, brightfield images were observed using an inverted microscope (IX71, OLYMPUS).

### Human clinical samples

Nine tissue specimens were collected from three patients with LC at various stages by the Affiliated Cancer Hospital of Inner Mongolia Medical University (Ref No.: YKD202101018). The specimens included pre-therapy samples (three tumor tissues and three adjacent normal tissues) and tissues from patients who exhibited a poor response to DDP therapy (three relapse tissues). All participants provided written informed consent, and the study was approved by the Ethics Committee of the Affiliated Cancer Hospital of Inner Mongolia Medical University (Ref No.: YKD202101018), in accordance with the Declarations of Helsinki.

### Immunofluorescence (IF) assay

Paraffin-embedded tissues were cut into 5-µm thick sections. Next, sections were stained with anti-IGF2BP2 antibody (No. ab124930, Abcam) and anti-CD133 antibody (No. PA5-38014, ThermoFisher SCIENTIFIC) overnight at 4 °C. Following this, the sections were probed with the secondary antibody (No. ab150077, Abcam). Finally, a fluorescence microscope (BX51, OLYMPUS) was used for observing images. The fluorescence semi-quantitative analysis was conducted with the Image Pro Plus software.

### Animal study

The animal study was conducted in compliance with the NIH Guidelines for the Care and Use of Laboratory Animals and performed in accordance with ARRIVE guidelines. BALB/c nude mice were obtained from Charles River and randomly divided into five groups (*n* = 3), A549 group, A549 + DDP group, A549/DDP group, A549/DDP + DDP group, A549/DDP + DDP + Lv-si-IGF2BP2 group. Each mouse in the A549, A549 + DDP, A549/DDP and A549/DDP + DDP groups was subcutaneously injected with A549 or A549/DDP cells (1*10^6^ cells) into the left flank. Additionally, each mouse in the A549/DDP + DDP + Lv-si-IGF2BP2 group was subcutaneously injected with Lv-si-IGF2BP2-transfected A549/DDP cells into the left flank. Mice in the DDP treatment groups were administered DDP (5 mg/kg) by intraperitoneal injection twice a week, as described previously [31]. Tumor sizes (volume = length × width^2^/2) were monitored weekly for 4 weeks. After treatment, the mice were anesthetized using 1% isoflurane inhalation and then sacrificed by cervical dislocation, after which tumor tissues were collected. Subsequently, paraffin-embedded tissues were cut into 5-µm thick sections. After that, sections were subjected to HE staining and then observed using a light microscope (SOPTOP OD630K, SUNNY HENGPING INSTRUMENT). All animal procedures were approved by the Committee of the Affiliated Cancer Hospital of Inner Mongolia Medical University (Ref No.: YKD202101018).

For the methylated RNA immunoprecipitation sequencing (MeRIP-seq) assay, mice were randomly divided into two groups: Lv-si-NC and Lv-si-IGF2BP2 groups. A549 cells transfected with either Lv-si-NC or Lv-si-IGF2BP2 were subcutaneously injected into the right flank of each mouse. After 4 weeks, the mice were anesthetized using 1% isoflurane inhalation and then sacrificed by cervical dislocation, after which the tumor tissues were then collected.

### M6A RNA methylation assay

The Redzol reagent (No. FTR-50, SBS Genetech Co., Ltd.) was used to extract total RNA from tumor tissues. Subsequently, the m6A levels of the mRNA were evaluated using the EpiQuik m6A RNA Methylation Quantification Kit (No. P-9005, Epigentek) in accordance with the manufacturer’s instructions. After that, the optical density (OD) at 450 nm was measured using a microplate reader (DNM-9602; PERLONG).

### Methylated RNA immunoprecipitation sequencing (MeRIP-seq)

The Redzol reagent was used to extract total RNA from tumor tissues. The isolated RNA samples were divided into two parts: immunoprecipitated (IP) and input parts. Next, RNA samples (IP parts) used for constructing the RIP Library were incubated with an anti-m6A antibody, and the complex were then bound to Dynabeads. After that, m6A-positive RNA was eluted from the m6A-Dynabeads. Meanwhile, RNA samples from the input parts were used for constructing the Input library. Upon completion of the library construction, the Illumina Hiseq X Ten was used for sequencing. The number of m6A peaks was calculated using the m6A Viewer software based on the sequencing data [32]. The DREME (http://meme-suite.org/tools/dreme) software was utilized to identify the m6A motif. UCSC snapshots of MeRIP-seq reads were utilized to visualize the indicated genes.

The sequencing data from the input samples served as transcriptome data. Correlation analysis was performed on the MeRIP-Seq data and the transcriptome data.

### Gene set enrichment analysis (GSEA)

Using the R package “clusterProfiler”, GSEA was performed on genes. The significantly enriched Gene Ontology [GO, including biological process (BP), molecular function (MF) and cell composition (CC)] terms and Kyoto Encyclopedia of Genes and Genomes (KEGG) pathways were identified according to the threshold value of |normalized enrichment score (NES)| > 1 and *p-*value < 0.05.

### Statistical analysis

Each experiment was independently repeated at least three times. Data were presented as mean ± standard deviation (SD). Differences between two groups were assessed using an unpaired Student’s t-test. Meanwhile, one‐way analysis of variance (ANOVA) or two-way ANOVA followed by Tukey test was employed for multiple comparisons. A p-value of < 0.05 indicates statistical significance.

## Results

### Diminished IGF2BP2 triggered A549 cell apoptosis and cell cycle arrest

**Fig. 1:**
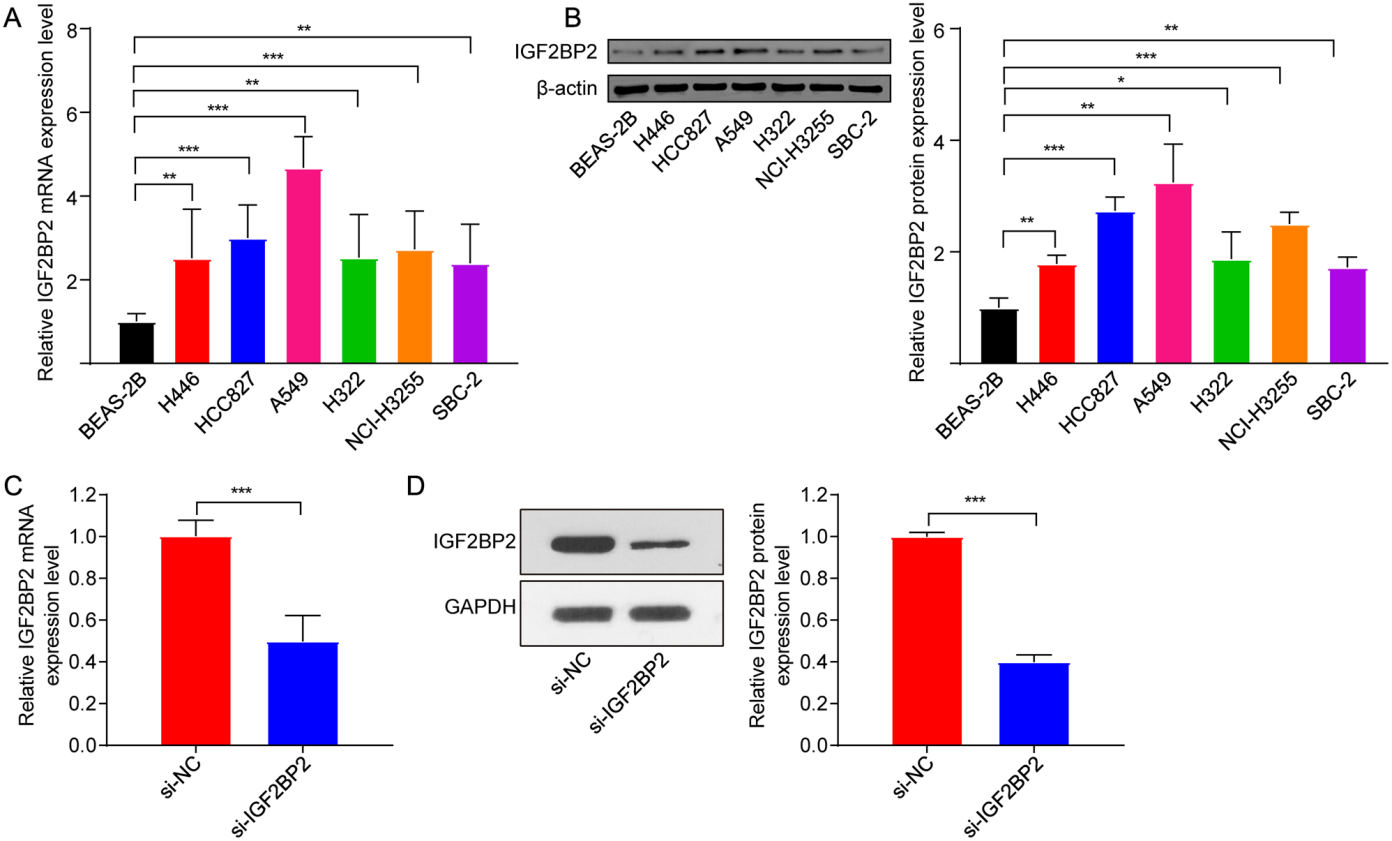
IGF2BP2 was increased in LC cells. **(A)** RT-qPCR and **(B)** western blot assays were applied to determine the mRNA and protein levels of IGF2BP2 in six LC cells. **(C)** RT-qPCR and **(D)** western blot assays were performed to assess the mRNA and protein levels of IGF2BP2 in A549 cell transfected with si-IGF2BP2. *P < 0.05; **P < 0.01; ***P < 0.001

IGF2BP2 has been identified as having oncogenic roles in human cancers [33]. Our results indicated that, compared to BEAS-2B cells, IGF2BP2 levels were significantly higher in LC cells, with A549 cells showing the highest expression of IGF2BP2 (Fig. 1A and B). Thus, A549 cells were selected for further experiments.

**Fig. 2:**
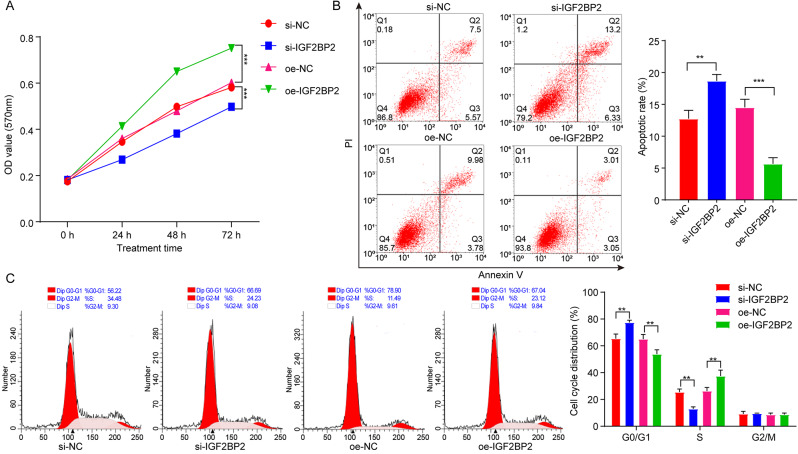
Deficiency of IGF2BP2 triggered A549 cell apoptosis and cell cycle arrest. A549 cells were transfected with si-IGF2BP2 or oe-IGF2BP2 plasmids. **(A)** The viability of A549 cells at 0, 24, 48 and 72 h were assessed using the MTT assay. **(B)** Cell apoptosis and **(C)** cell cycle distribution of A549 cells were evaluated by flow cytometry. **P < 0.01; ***P < 0.001

To explore the impact of IGF2BP2 in lung cancer, A549 cells were transfected with si-IGF2BP2 to downregulate IGF2BP2 levels (Fig. 1C and D). Additionally, downregulation of IGF2BP2 obviously repressed A549 cell viability and triggered cell apoptosis; conversely, IGF2BP2 overexpression displayed opposite effects (Fig. 2A and B). Moreover, IGF2BP2 deficiency strongly increased the number of cells in the G0/G1 phase, but decreased the number of cells in the S phase (Fig. 2C), leading to cell cycle arrest. Conversely, IGF2BP2 overexpression facilitated the transition of cells from the G0/G1 phase to the S phase in A549 cells (Fig. 2C). Collectively, downregulation of IGF2BP2 could lead to apoptosis and cell cycle arrest in A549 cells .

### Downregulation of IGF2BP2 repressed DDP resistance in A549/DDP cells

**Fig. 3:**
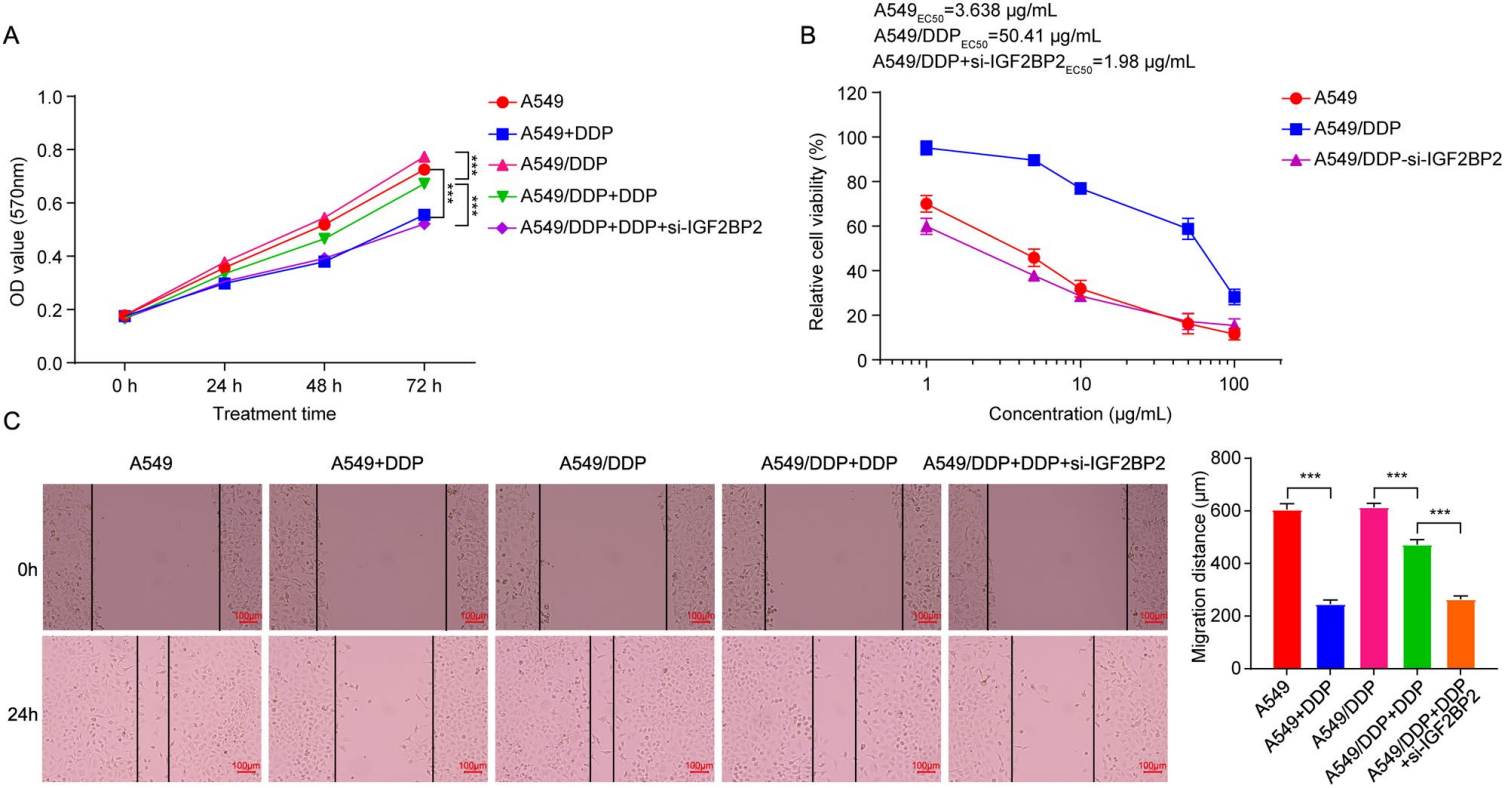
Downregulation of IGF2BP2 augmented the anti-proliferative and anti-migratory effects of DDP on A549/DDP cells. (**A**) A549 cells were treated with DDP, and A549/DDP cells were treated with DDP, or DDP + si-IGF2BP2 for 0, 24, 48 and 72 h. (**B**) A549, A549/DDP and si-IGF2BP2-transfected A549/DDP cells were treated with DDP (0, 1, 5, 10, 50 or 100 µg/mL) for 48 h. Cell viability was assessed using the MTT assay. (**C**) A549 cells were treated with DDP, and A549/DDP cells were treated with DDP or DDP + si-IGF2BP2 for 0 and 24 h. Cell migration was evaluated using the wound healing assay (scale bar, 100 μm). ***P < 0.001

Next, we explored the role of IGF2BP2 in the resistance of LC cells to DDP. MTT results showed that DDP remarkably reduced A549 cell viability by approximately 24% following a 72-hour treatment (Fig. 3A). However, treated A549/DDP cells with DDP for 72 h resulted in a mere 13% reduction in the viability of A549/DDP cells; as expected, the combination of DDP and si-IGF2BP2 led to a 34% decrease in cell viability, suggesting that the silencing of IGF2BP2 further intensified the cytotoxic effects of DDP on A549/DDP cells (Fig. 3A). The half maximal effective concentration (EC_50_) of DDP in A549 cells was determined to be 3.638 µg/ml (Fig. 3B). In the presence of si-IGF2BP2, the EC50 value for DDP in A549 cells was decreased to 0.982 µg/ml (Figure S1). Additionally, the EC_50_ for DDP in A549/DDP cells was found to be 50.41 µg/ml, indicating that these cells exhibit resistance to DDP (Fig. 3B). Meanwhile, when combined with si-IGF2BP2, the EC50 value for DDP in A549/DDP cells was reduced to 1.98 µg/ml (Fig. 3B).

**Fig. 4:**
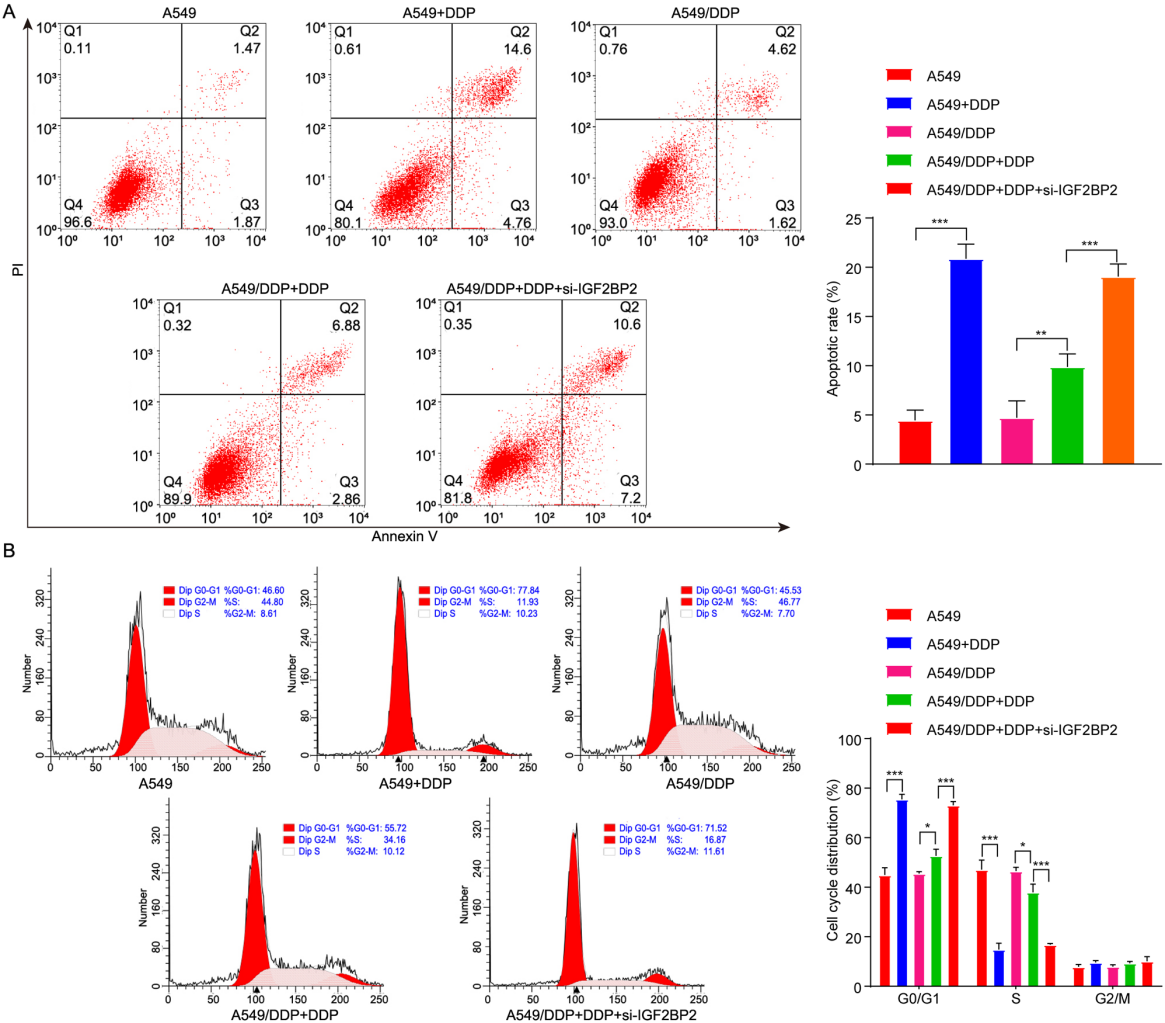
Downregulation of IGF2BP2 enhanced the pro-apoptotic effect of DDP on A549/DDP cells. A549 cells were treated with DDP, and A549/DDP cells were treated with DDP or DDP + si-IGF2BP2 for 48 h. **(A)** Cell apoptosis and **(B)** cell cycle distribution were evaluated by flow cytometry. *P < 0.05;  **P < 0.01; ***P < 0.001

Furthermore, DDP significantly inhibited cell migration, increased cell apoptosis and induced cell cycle arrest at the G0/G1 phase in A549/DDP cells (Figs. 3C and 4A and B). As expected, compared to DDP treatment alone, the combination of DDP and si-IGF2BP2 further enhanced these effects (Figs. 3C and 4A and B), suggesting that downregulation of IGF2BP2 could enhance the anti-migratory, pro-apoptotic and cell cycle-arresting effects of DDP in A549/DDP cells. To sum up, IGF2BP2 knockdown could promote the sensitivity of A549/DDP cells to DDP.

### IGF2BP2 was apparently elevated in relapsed lung cancer tissues

**Fig. 5:**
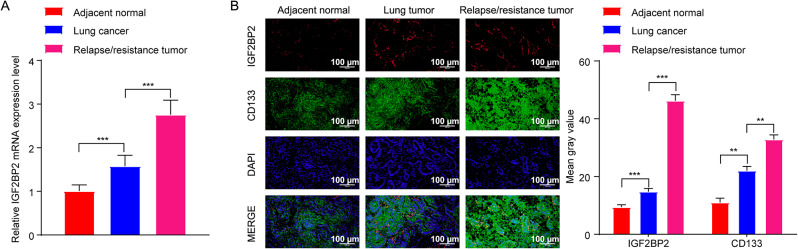
IGF2BP2 was apparently elevated in relapsed LC tissues. **(A)** RT-qPCR was performed to assess IGF2BP2 levels in adjacent normal tissues, tumor tissues and relapse/resistant tumor tissues (poor response to DDP therapy). **(B)** IF staining assay was used to determine IGF2BP2 and CD133 levels in adjacent normal tissues, tumor tissues and relapse/resistant tumor tissues (scale bar, 100 μm). **P < 0.01; ***P < 0.001

The results of RT-qPCR and IF staining results showed that, compared to normal tissues, IGF2BP2 levels were notably elevated in LC tissues and relapsed/resistant LC tissues (Fig. 5A and B). Meanwhile, compared to LC tissues, IGF2BP2 levels were markedly increased in relapsed/resistant LC tissues (Fig. 5A and B). Moreover, CD133, a marker for tumor initiation and relapse, was found to be significantly higher in relapsed/resistant LC tissues than in LC tissues (Fig. 5B). Therefore, elevated levels of IGF2BP2 may be related to tumor relapse after DDP therapy in LC.

### Downregulation of IGF2BP2 reduced the resistance of A549/DDP cells to DDP in vivo

**Fig. 6:**
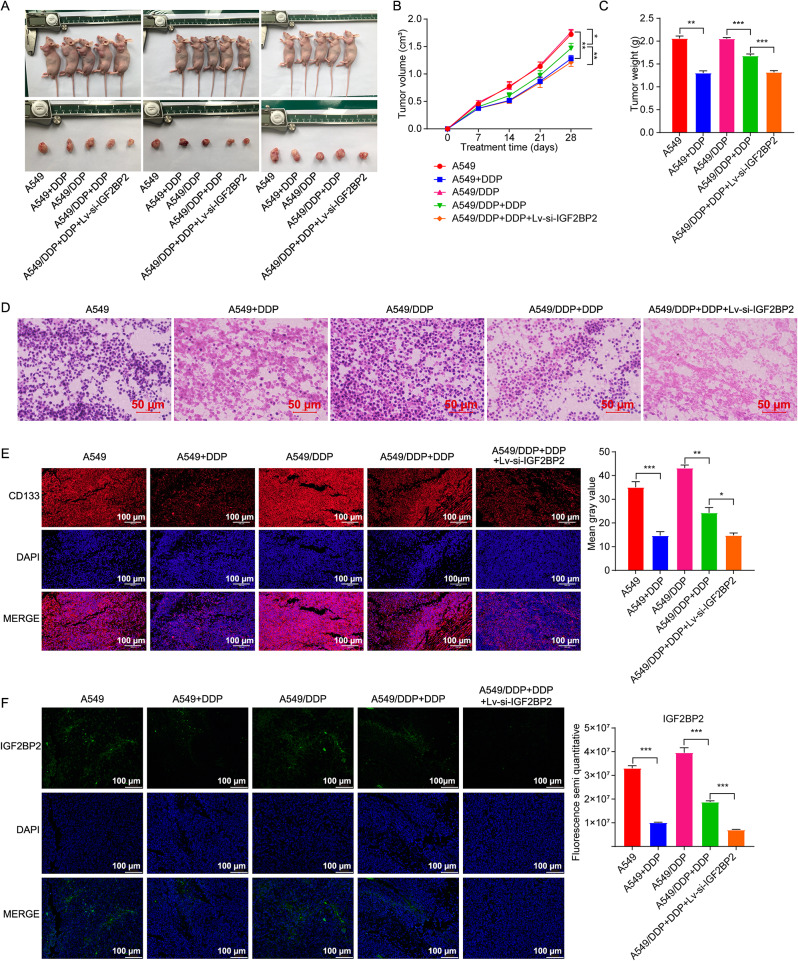
Downregulation of IGF2BP2 reduced the resistance of A549/DDP cells to DDP *in vivo.***(A)** Photographs of animals (upper panel) and xenograft tumors (lower panel). **(B)** Tumor volume and **(C)** tumor weight. **(D)** H&E staining assay was done to observe pathological changes of tumor tissues (scale bar, 50 μm). **(E**,** F)** IF staining assay was done to assess CD133 and IGF2BP2 levels in tumor tissues (scale bar, 100 μm). *P < 0.05; **P < 0.01; ***P < 0.001

We then explored the impact of IGF2BP2 in the regulation of drug resistance using a murine xenograft mouse model of A549/DDP cells. DDP treatment obviously decreased tumor volume and weight in A549/DDP xenograft mice (Fig. 6A, B and C). Additionally, IGF2BP2 deficiency led to an additional reduction in both tumor volume and weight compared to the A549/DDP + DDP group (Fig. 6A, B and C). Moreover, HE staining revealed obvious necrosis in the tumor tissues of the A549/DDP + DDP group; with even more pronounced necrosis observed following the silencing of IGF2BP2 (Fig. 6D). Furthermore, compared to A549/DDP + DDP group, IGF2BP2 deficiency further reduced the levels of CD133 and IGF2BP2 in tumor tissues from A549/DDP xenograft mice (Fig. 6E and F). Collectively, downregulation of IGF2BP2 could reduce the resistance of A549/DDP cells to DDP in vivo.

### IGF2BP2 affected m6A methylation levels in LC

**Fig. 7:**
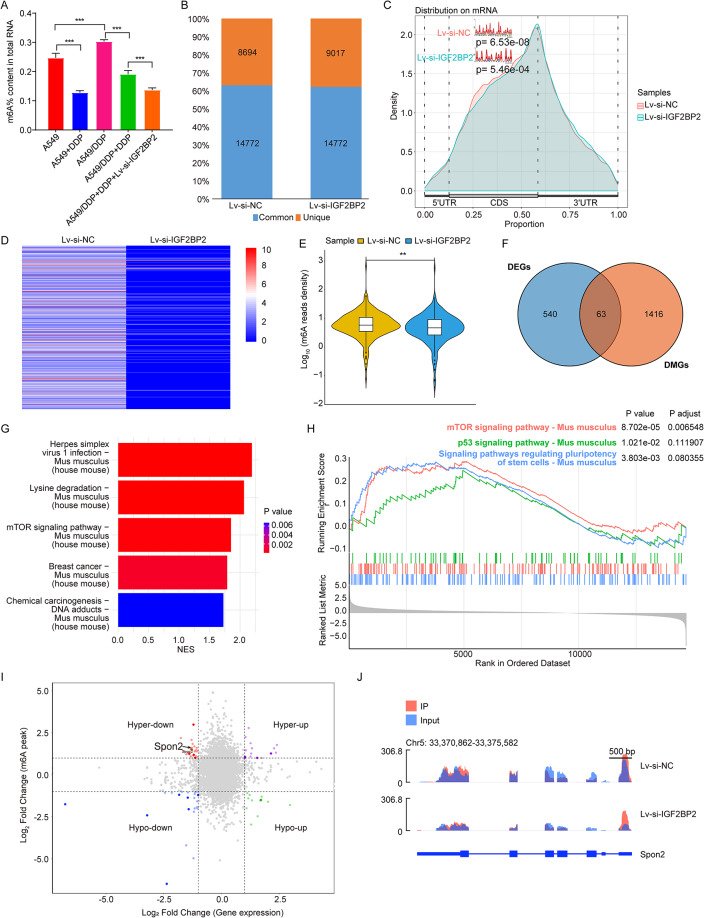
IGF2BP2 affected m6A methylation modification in LC. **(A)** The RNA m6A methylation levels in tumor tissues from A549 or A549/DDP xenograft mice were evaluated. ***P < 0.001. **(B)** The number of m6A peaks was calculated using the m6A Viewer software. **(C)** Normalized distribution of m6A peaks and identification of m6A motif. **(D)** Heatmap showed the levels of m6a modified genes in two group. **(E)** The normalized m6A reads density of common m6A peak was shown in Violin plot. **(F)** Venn diagram was used for identifying overlapping genes between differentially expressed genes (DEGs) and differentially methylated genes (DMGs). **(G)** Top 5 signalings from GSEA-KEGG analysis. **(H)** The “Signaling pathways regulating pluripotency of stem cells”, “mTOR signaling pathway”, and “p53 signaling pathway” were related to 63 overlapping genes. **(I)** The quadrantal diagram graph of transcription and m6A methylation levels using MeRIP-seq data. **(J)** UCSC snapshots of m6A-seq reads was used for visualizing Spon2

It has been shown that IGF2BP2 is a critical regulator of m6A methylation [34]. Meanwhile, our results showed that compared to the A549/DDP group, DDP treatment obviously declined the m6A methylation levels in tumor tissues (Fig. 7A). Notably, IGF2BP2 deficiency led to a further reduction in m6A methylation levels compared to the A549/DDP + DDP group (Fig. 7A). These data showed that IGF2BP2 has the ability to suppress the m6A methylation levels in LC.

Thus, to identify the m6A modification targets in LC, the MeRIP-seq was performed. A total of 23,466 and 23,789 m6A peaks were detected in Lv-si-NC and Lv-si-IGF2BP2 groups respectively, with 14,772 peaks identified in both two groups (Fig. 7B). Additionally, the m6A consensus motif AAAAAAAAAAAAAAAAAAAAA was enriched in the detected peaks in both groups (Fig. 7C). Compared to the Lv-si-NC group, most of m6A-modified genes and m6A abundance of common peaks were markedly decreased in tumor tissues in the Lv-si-IGF2BP2 group (Fig. 7D and E). Furthermore, 540 differentially expressed genes (DEGs) and 1,416 differentially methylated genes (DMGs) were identified between the Lv-si-NC and Lv-si-IGF2BP2 groups (Table S2, S3). Meanwhile, there were 63 overlapping genes between DEGs and DMGs (Fig. 7F and Table S4). Subsequently, GSEA analysis was performed on these overlapping genes. GSEA-GO analysis revealed that these overlapping genes were significantly enriched in 1,644 processes such as “kinase activity”, “histone modification” and “microtubule organizing center” (Figure S2A and Table S5). GSEA-KEGG results showed that these overlapping genes were related to 68 pathways, such as “Herpes simplex virus 1 infection”, “Lysine degradation”, “mTOR signaling pathway”, “Signaling pathways regulating pluripotency of stem cells” and “p53 signaling pathway” (Fig. 7G and H, S2B and Table S5).

Based on the correlation analysis between methylation and transcriptome level, the m6A levels of Spon2 gene were significantly increased, whereas the mRNA levels of Spon2 were notably reduced in the Lv-si-IGF2BP2 group, compared to the Lv-si-NC group (Fig. 7I). Meanwhile, the m6A peaks of Spon2 gene in Lv-si-NC and Lv-si-IGF2BP2 groups were displayed in Fig. 7J. Collectively, downregulation of IGF2BP2 could decrease Spon2 levels in tumor tissues via m6A RNA methylation.

### Downregulation of IGF2BP2 repressed DDP resistance in A549/DDP cells via downregulation of Spon2

**Fig. 8:**
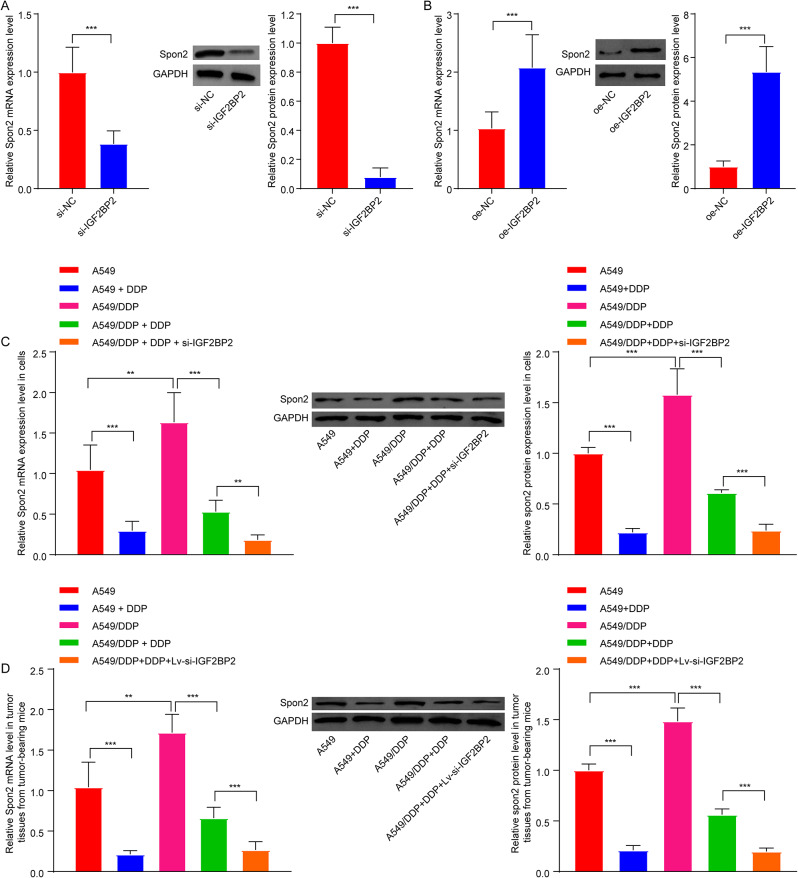
Downregulation of IGF2BP2 declined Spon2 expression in LC. A549 cells were transfected with **(A)** si-IGF2BP2 or **(B)** oe-IGF2BP2 plasmids. RT-qPCR and western blot assays were conducted to assess Spon2 levels in A549 cells. **(C)** A549 cells were treated with DDP, and A549/DDP cells were treated with DDP or DDP + si-IGF2BP2. RT-qPCR and western blot assays were conducted to assess Spon2 levels in cells. **(D)** RT-qPCR and western blot assays were conducted to determine Spon2 levels in tumor tissues from A549 or A549/DDP xenograft mice. **P < 0.01; ***P < 0.001

**Fig. 9:**
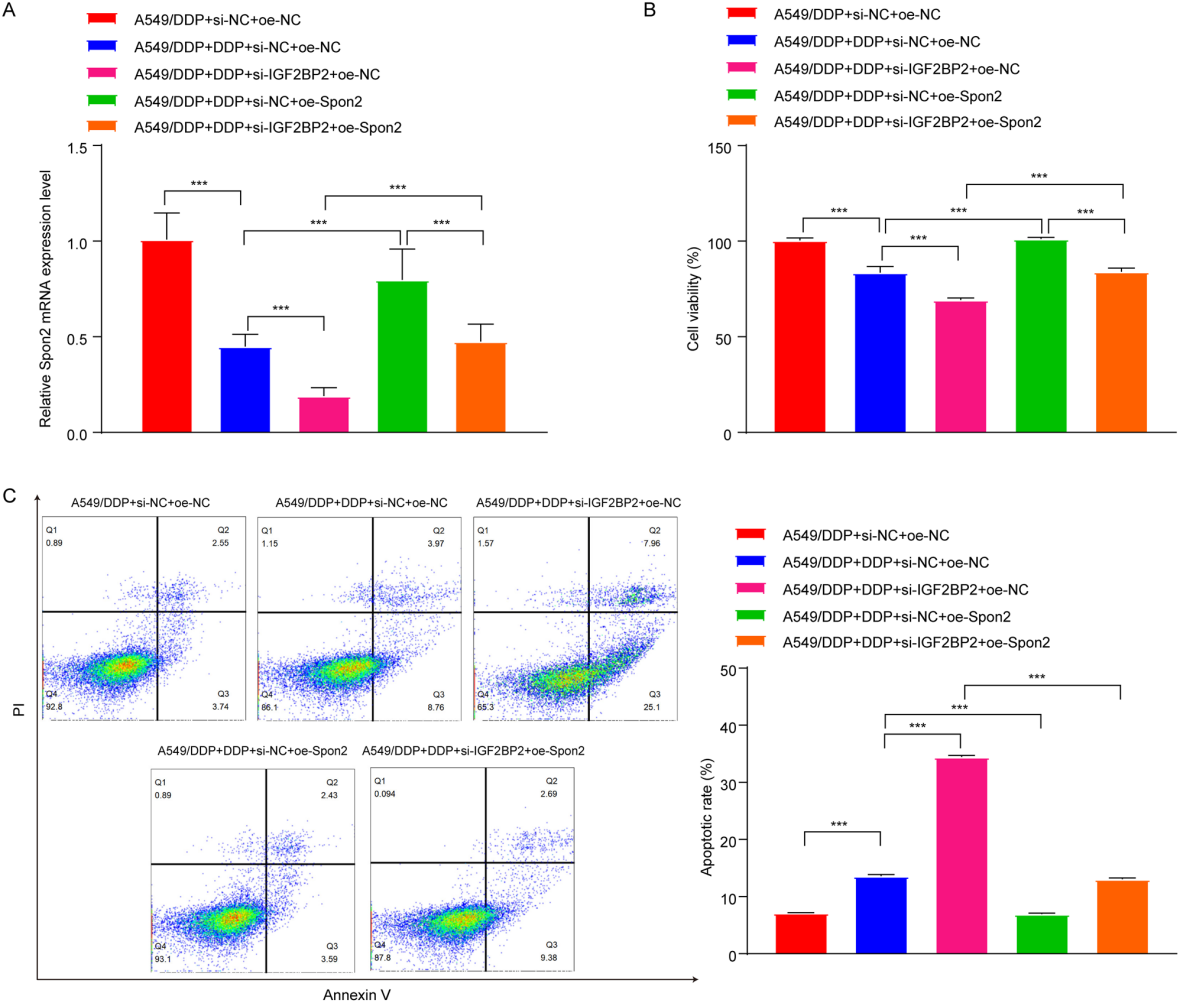
Downregulation of IGF2BP2 repressed DDP resistance in A549/DDP cells via downregulation of Spon2. A549/DDP cells were treated with DDP, DDP + si-IGF2BP2, DDP + oe-Spon2 or DDP + si-IGF2BP2 + oe-Spon2. **(A)** RT-qPCR assay was conducted to assess Spon2 levels in cells. **(B**) MTT, **(C)** flow cytometry assays were applied to evaluate cell viability and apoptosis. ***P < 0.001

Next, we investigated whether IGF2BP2 could impact DDP resistance in LC via modulation of Spon2. The results of RT-qPCR and western blot assays showed that downregulation of IGF2BP2 led to a significant decrease in Spon2 expression in A549 cells, whereas overexpression of IGF2BP2 resulted in an increase in Spon2 expression in A549 cells (Fig. 8A and B). Additionally, compared to the A549 group, Spon2 levels were obviously elevated in A549/DDP cells and in tumor tissues from A549/DDP xenograft mice (Fig. 8C and D). Significantly, DDP treatment caused a significant reduction in Spon2 levels in both A549/DDP cells and tumor tissues from A549/DDP xenograft mice, compared to the A549/DDP group; meanwhile, IGF2BP2 deficiency further reduced Spon2 expression (Figs. 8C and D and 9A). Conversely, compared to the DDP + si-IGF2BP2 group, overexpression of Spon2 remarkably elevated Spon2 levels in DDP + si-IGF2BP2-treated A549/DDP cells (Fig. 9A). Furthermore, compared to the DDP + si-NC + pcDNA3.1-NC group, IGF2BP2 deficiency remarkably enhanced the anti-proliferative and pro-apoptotic effects of DDP in A549/DDP cells; however, these effects were notably reversed by Spon2 overexpression (Fig. 9B and C). Collectively, our findings suggested that downregulation of IGF2BP2 was able to overcome DDP resistance in A549/DDP cells via downregulation of Spon2.

## Discussion

Drug resistance has long posed a significant challenge in LC [35]. Resistance to chemotherapy, and subsequent relapse are the main reasons leading to poor survival rates in LC patients [36]. Treatment failure and tumor relapse are common after DDP treatment in a substantial proportion of DDP-based treatment patients [37]. Consequently, it is essential to find effective strategies to overcome acquired resistance to DDP in LC patients. Our results revealed that deficiency of IGF2BP2 could overcome DDP resistance in LC in vitro and in vivo.

Compared to the A549 group, m6A RNA modification levels were obviously elevated in the A549/DDP group, suggesting that m6A methylation is associated with DDP resistance in LC. Numerous studies have confirmed that m6A methylation exerts important roles in modulating cancer progression and drug resistance [38, 39]. M6A methyltransferase METTL3 has been reported to contribute to oxaliplatin resistance by stabilizing PARP1 mRNA in CD133 + gastric cancer stem cells [40]. Meanwhile, METTL3 could increase AKT levels in NSCLC cells, thereby reducing sensitivity to DDP [41]. ALKBH5, an m6A demethylase, is highly expressed in DDP-resistant epithelial ovarian cancer (EOC) cells; and forced expression of ALKBH5 could confer resistance to DDP in EOC cells [42]. YTHDF1, an m6A reader protein, is over-expressed in gastrointestinal cancers, and elevated levels of YTHDF1 have been associated with chemoresistance [43]. Furthermore, IGF2BP2, another m6A reader protein, has been shown to enhance chemoresistance in glioma cells [28]. However, no studies have reported the role of IGF2BP2 in DDP-resistant LC. In the present study, we observed high levels of IGF2BP2 in relapsed/resistant LC tissues from patients compared to LC tissues obtained from non-resistant patients. These results revealed a potential role for IGF2BP2 in facilitating DDP resistance in LC.

The IGF2BP family consists of three members including IGF2BP1, IGF2BP2, and IGF2BP3 [44], all of which are implicated in the drug resistance of human cancers [45–47]. IGF2BP1 knockdown has been shown to reduce resistance to chemotherapeutic drugs (e.g. 5-fluorouracil, irinotecan, and oxaliplatin) in colorectal cancer (CRC) cells [45]. IGF2BP2 could facilitate DDP resistance in CRC via enhancing the stabilization of long non-coding RNA taurine up-regulated gene 1 [46]. Additionally, IGF2BP3-mediated m6A modification could promote the stabilization of TMA7 mRNA, thereby contributing to cancer progression and DDP resistance in laryngeal cancer [47]. In the present research, we found that reduced expression of IGF2BP2 could further enhance the anti-proliferative, anti-migratory, and pro-apoptotic effects of DDP on A549/DDP cells. Thus suggests that IGF2BP2 downregulation could effectively overcome DDP resistance in DDP-resistant LC cells.

DDP can crosslink DNA and inhibit DNA synthesis, ultimately leading to cell death [48]. However, chemoresistance often inevitably appears during DDP treatment [49]. In the present study, we found that DDP induced cytotoxicity, cell apoptosis and cell cycle arrest in A549/DDP cells, with these effects greatly augmented by IGF2BP2 knockdown. These findings demonstrated that IGF2BP2 knockdown could overcome DDP resistance in DDP-resistant LC cells by triggering cell cycle arrest and apoptosis.

M6A RNA methylation has been implicated in the progression of several cancers, including LC [50, 51]. Mechanistically, m6A methylation plays a crucial role in modulating gene expression at the post-transcriptional level by influencing mRNA stability, splicing, and translation [51–53]. As an m6A “reader”, IGF2BP2 has been shown to enhance the stability of SLC7A11 mRNA through m6A modification, thereby conferring ferroptosis resistance to hepatoblastomas [54]. Our results showed that compared to the Lv-si-NC group, downregulation of IGF2BP2 remarkably elevated the m6A levels of Spon2, but reduced the mRNA levels of Spon2 in tumor tissues of mice, suggesting that downregulation of IGF2BP2 could reduce Spon2 levels in tumor tissues via the modulation of m6A methylation. Some studies have shown that the presence of m6A modification can result in mRNA degradation [55, 56]. Li et al. found that IGF2BP2 can enhance the stability of SOX2 transcripts, thereby preventing their degradation via an m6A-IGF2BP2-dependent mechanism [24]. Our results indicated an increased m6A level of Spon2 in the Lv-si-IGF2BP2 group. Thus, we hypothesize that IGF2BP2 downregulation results in elevated m6A levels of Spon2, which subsequently causes mRNA degradation and ultimately leads to reduced mRNA levels of Spon2; however, the exact mechanism of this process warrants further investigation. Both Spon2 and IGF2BP2 have been implicated in the progression and metastasis of human cancers, including LC [27, 57, 58]. Thus, we speculated that downregulation of IGF2BP2 may inhibit LC progression through downregulating Spon2 in an m6A-dependent manner. Notably, our study is the first to show the relationship between the Spon2 and IGF2BP2 in LC.

Furthermore, our results demonstrated that IGF2BP2 deficiency could reduce Spon2 expression levels in the A549/DDP + DDP group. Moreover, IGF2BP2 deficiency enhanced the pro-apoptotic effects of DDP in A549/DDP cells, whereas these changes were reversed by Spon2 overexpression, suggesting that the knockdown of IGF2BP2 could potentially overcome DDP resistance in A549/DDP cells through the downregulation of Spon2. Collectively, our data showed that the knockdown of IGF2BP2 could prevent DDP resistance in LC by downregulating Spon2 in an m6A-dependent manner. However, this study focused exclusively on the impact of IGF2BP2 siRNA on A549 and A549/DDP cells. Further research is needed to validate the role of IGF2BP2 siRNA in DDP resistance across other LC cell lines and DDP-resistant LC cells in future studies. Furthermore, a therapeutic strategy that combines an IGF2BP2 inhibitor with DDP warrants exploration in subsequent trials, which may provide further insights into the application of IGF2BP2 in LC.

Huang et al. demonstrated that Spon2 overexpression could facilitate colorectal cancer progression through enhancing the infiltration of M2 macrophages [59]. Research has indicated that M2-like tumor-associated macrophages (TAMs) play important roles in tumor progression and acquired drug resistance [60]. M2-like TAMs can contribute to chemotherapy resistance by secreting cytokines or activating anti-apoptotic pathways [61]. Thus, we hypothesize that the IGF2BP2-Spon2 axis may influence DDP resistance in LC by promoting M2 macrophage polarization; however, this hypothesis requires further investigation.

## Conclusion

Together, downregulation of IGF2BP2 could inhibit DDP resistance in LC through downregulating Spon2 gene expression in an m6A-dependent manner. These findings may provide a new strategy for overcoming DDP resistance in LC.

## Electronic supplementary material

Below is the link to the electronic supplementary material.

Supplementary Material 1: Figure S1: Knockdown of IGF2BP2 augmented DDP cytotoxicity on A549 cells. si-IGF2BP2-transfected A549 cells were treated with DDP (0, 1, 5, 10, 50, or 100 μg/mL) for 48 h. Cell viability was assessed using the MTT assay.

Supplementary Material 2 Figure S2: Functional enrichment analyses. (A) GSEA-GO and (B) GSEA-KEGG enrichment analysis.

Supplementary Material 3

Supplementary Material 4: Table S1: The sequences of si-IGF2BP2, oe-IGF2BP2, oe-Spon2.

Supplementary Material 5: Table S2: 540 differentially expressed genes (DEGs) between Lv-si-NC and Lv-si-IGF2BP2 groups.

Supplementary Material 6: Table S3: 1416 differentially methylated genes (DMGs) between Lv-si-NC and Lv-si-IGF2BP2 groups.

Supplementary Material 7: Table S4: 63 overlapping genes betweeen DEGs and DMGs.

Supplementary Material 8: Table S5 GSEA analysis.

## Data Availability

The datasets used and/or analysed during the current study are available from the corresponding author on reasonable request.

## References

[1] Zhu Y, Cui Y, Zheng X, et al. Small-cell lung cancer brain metastasis: from molecular mechanisms to diagnosis and treatment. Biochim et Biophys acta Mol Basis Disease. 2022;1868(12):166557. 10.1016/j.bbadis.2022.166557 36162624

[2] Fan XX, Wu Q. Decoding Lung Cancer at single-cell level. Front Immunol. 2022;13:883758. 35677034 10.3389/fimmu.2022.883758 PMC9167930

[3] Pirlog R, Chiroi P, Rusu I et al. (2022) Cellular and Molecular Profiling of Tumor Microenvironment and Early-Stage Lung Cancer. Int J Mol Sci 23(10). 10.3390/ijms23105346 PMC9140615 35628157

[4] Meijer JJ, Leonetti A, Airo G, et al. Small cell lung cancer: novel treatments beyond immunotherapy. Semin Cancer Biol. 2022;86(Pt 2):376–85. 35568295 10.1016/j.semcancer.2022.05.004

[5] Wang WZ, Shulman A, Amann JM, et al. Small cell lung cancer: subtypes and therapeutic implications. Semin Cancer Biol. 2022;86(Pt 2):543–54. 35398266 10.1016/j.semcancer.2022.04.001

[6] Kim JS, Kim EJ, Jang JG et al. (2023) Low diffusion capacity predicts poor prognosis in extensive stage small cell lung cancer: a single-center analysis of 10 years. J Cancer Res Clin Oncol. 10.1007/s00432-023-04686-2 PMC10374757 36912944

[7] Li J, Zhu H, Sun L, et al. Prognostic value of site-specific metastases in lung cancer: a population based study. J Cancer. 2019;10(14):3079–86. 31289577 10.7150/jca.30463 PMC6603375

[8] Samarth N, Gulhane P, Singh S. Immunoregulatory framework and the role of miRNA in the pathogenesis of NSCLC - A systematic review. Front Oncol. 2022;12:1089320. 36620544 10.3389/fonc.2022.1089320 PMC9811680

[9] Zou J, Guo S, Xiong MT, et al. Ageing as key factor for distant metastasis patterns and prognosis in patients with extensive-stage small cell Lung Cancer. J Cancer. 2021;12(6):1575–82. 33613744 10.7150/jca.49681 PMC7890308

[10] Lemjabbar-Alaoui H, Hassan OU, Yang YW, et al. Lung cancer: Biology and treatment options. Biochim Biophys Acta. 2015;1856(2):189–210. 26297204 10.1016/j.bbcan.2015.08.002 PMC4663145

[11] Johnson ML, Cho BC, Luft A, et al. Durvalumab with or without Tremelimumab in Combination with Chemotherapy as First-Line therapy for metastatic non-small-cell lung Cancer: the Phase III POSEIDON Study. J Clin Oncol. 2023;41(6):1213–27. 36327426 10.1200/JCO.22.00975 PMC9937097

[12] Robert F, Omura GA, Birch R, et al. Randomized phase III comparison of three doxorubicin-based chemotherapy regimens in advanced non-small cell lung cancer: a Southeastern Cancer Study Group trial. J Clin Oncol. 1984;2(5):391–5. 6374050 10.1200/JCO.1984.2.5.391

[13] Kryczka J, Kryczka J, Czarnecka-Chrebelska KH et al. (2021) Molecular mechanisms of Chemoresistance Induced by Cisplatin in NSCLC Cancer Therapy. Int J Mol Sci 22(16). 10.3390/ijms22168885 PMC8396273 34445588

[14] Ding L, Li L, Tang Z. Cisplatin resistance and malignant behaviors of lung cancer cells are promoted by circ_0002360 via targeting mir-6751-3p to regulate the expression of ZNF300. Thorac Cancer. 2022;13(7):986–96. 35166026 10.1111/1759-7714.14342 PMC8977170

[15] Zhao M, Wang T, Hui Z. Aspirin overcomes cisplatin resistance in lung cancer by inhibiting cancer cell stemness. Thorac Cancer. 2020;11(11):3117–25. 32991066 10.1111/1759-7714.13619 PMC7605995

[16] Jafarzadeh E, Montazeri V, Aliebrahimi S, et al. Combined regimens of cisplatin and metformin in cancer therapy: a systematic review and meta-analysis. Life Sci. 2022;304:120680. 35662589 10.1016/j.lfs.2022.120680

[17] Huang Y, Lei L, Liu Y. Propofol improves sensitivity of Lung Cancer cells to cisplatin and its mechanism. Med Sci Monitor: Int Med J Experimental Clin Res. 2020;26:e919786. 10.12659/MSM.919786 PMC7142322 32225124

[18] Teng PC, Liang Y, Yarmishyn AA et al. (2021) RNA Modifications and Epigenetics in Modulation of Lung Cancer and Pulmonary Diseases. Int J Mol Sci. 22(19). 10.3390/ijms221910592 PMC8508636 34638933

[19] Bajbouj K, Al-Ali A, Ramakrishnan RK et al. (2021) Histone Modification in NSCLC: Molecular Mechanisms and Therapeutic Targets. Int J Mol Sci. 22(21). 10.3390/ijms222111701 PMC8584007 34769131

[20] Roundtree IA, Evans ME, Pan T, et al. Dynamic RNA modifications in Gene expression regulation. Cell. 2017;169(7):1187–200. 28622506 10.1016/j.cell.2017.05.045 PMC5657247

[21] Zhao W, Qi X, Liu L, et al. Epigenetic regulation of m(6)a modifications in Human Cancer. Mol Ther Nucleic Acids. 2020;19:405–12. 31887551 10.1016/j.omtn.2019.11.022 PMC6938965

[22] An Y, Duan H. The role of m6A RNA methylation in cancer metabolism. Mol Cancer. 2022;21(1):14. 35022030 10.1186/s12943-022-01500-4 PMC8753874

[23] Jiang X, Liu B, Nie Z, et al. The role of m6A modification in the biological functions and diseases. Signal Transduct Target Ther. 2021;6(1):74. 33611339 10.1038/s41392-020-00450-x PMC7897327

[24] Li T, Hu PS, Zuo Z, et al. METTL3 facilitates tumor progression via an m(6)A-IGF2BP2-dependent mechanism in colorectal carcinoma. Mol Cancer. 2019;18(1):112. 31230592 10.1186/s12943-019-1038-7 PMC6589893

[25] Li B, Zhu L, Lu C, et al. circNDUFB2 inhibits non-small cell lung cancer progression via destabilizing IGF2BPs and activating anti-tumor immunity. Nat Commun. 2021;12(1):295. 33436560 10.1038/s41467-020-20527-z PMC7804955

[26] Zhou Z, Zhu T, Chen S, et al. Systematic analysis of the expression Profile and Prognostic significance of the IGF2BP Family in Lung Adenocarcinoma. Curr Cancer Drug Targets. 2022;22(4):340–50. 35232349 10.2174/1568009622666220301145013

[27] Han L, Lei G, Chen Z, et al. IGF2BP2 regulates MALAT1 by serving as an N6-Methyladenosine reader to Promote NSCLC Proliferation. Front Mol Biosci. 2021;8:780089. 35111811 10.3389/fmolb.2021.780089 PMC8802805

[28] Han J, Yu X, Wang S, et al. IGF2BP2 induces U251 Glioblastoma Cell Chemoresistance by inhibiting FOXO1-Mediated PID1 expression through stabilizing lncRNA DANCR. Front Cell Dev Biol. 2021;9:659228. 35141227 10.3389/fcell.2021.659228 PMC8819069

[29] Wu EY, Huang LP, Bao JH. Mir-96-5p regulates cervical cancer cell resistance to cisplatin by inhibiting lncRNA TRIM52-AS1 and promoting IGF2BP2. Kaohsiung J Med Sci. 2022;38(12):1178–89. 36354205 10.1002/kjm2.12593 PMC11896587

[30] Livak KJ, Schmittgen TD. Analysis of relative gene expression data using real-time quantitative PCR and the 2(-Delta Delta C(T)) method. Methods. 2001;25(4):402–8. 11846609 10.1006/meth.2001.1262

[31] Huang G, Lou T, Pan J, et al. MiR-204 reduces cisplatin resistance in non-small cell lung cancer through suppression of the caveolin-1/AKT/Bad pathway. Aging. 2019;11(7):2138–50. 30981205 10.18632/aging.101907 PMC6503872

[32] Antanaviciute A, Baquero-Perez B, Watson CM, et al. m6aViewer: software for the detection, analysis, and visualization of N(6)-methyladenosine peaks from m(6)A-seq/ME-RIP sequencing data. RNA. 2017;23(10):1493–501. 28724534 10.1261/rna.058206.116 PMC5602108

[33] Xu X, Yu Y, Zong K, et al. Up-regulation of IGF2BP2 by multiple mechanisms in pancreatic cancer promotes cancer proliferation by activating the PI3K/Akt signaling pathway. J Exp Clin Cancer Res. 2019;38(1):497. 31852504 10.1186/s13046-019-1470-y PMC6921559

[34] Hu Y, Chen J, Liu M et al. (2022) IGF2BP2 serves as a core m6A regulator in head and neck squamous cell carcinoma. Biosci Rep 42(11). 10.1042/BSR20221311 PMC9653096 36281789

[35] Cui X, Zhang B, Li B, et al. Circular RNA circ_0002360 regulates the taxol resistance and malignant behaviors of Taxol-resistant non-small cell lung cancer cells by microRNA-585-3p-dependent modulation of G protein regulated inducer of neurite outgrowth 1. Bioengineered. 2022;13(4):9070–85. 35293280 10.1080/21655979.2022.2053803 PMC9162002

[36] Pan Z, Liu H, Chen J. [Lung Cancer stem-like cells and Drug Resistance]. Zhongguo Fei Ai Za Zhi. 2022;25(2):111–7. 35224964 10.3779/j.issn.1009-3419.2022.102.02 PMC8913289

[37] Galluzzi L, Vitale I, Michels J, et al. Systems biology of cisplatin resistance: past, present and future. Cell Death Dis. 2014;5(5):e1257. 24874729 10.1038/cddis.2013.428 PMC4047912

[38] Shriwas O, Mohapatra P, Mohanty S, et al. The impact of m6A RNA modification in Therapy Resistance of Cancer: implication in Chemotherapy, Radiotherapy, and Immunotherapy. Front Oncol. 2020;10:612337. 33718113 10.3389/fonc.2020.612337 PMC7947626

[39] Chen Y, Lin Y, Shu Y, et al. Interaction between N(6)-methyladenosine (m(6)A) modification and noncoding RNAs in cancer. Mol Cancer. 2020;19(1):94. 32443966 10.1186/s12943-020-01207-4 PMC7243333

[40] Li H, Wang C, Lan L, et al. METTL3 promotes oxaliplatin resistance of gastric cancer CD133 + stem cells by promoting PARP1 mRNA stability. Cell Mol Life Sci. 2022;79(3):135. 35179655 10.1007/s00018-022-04129-0 PMC11072755

[41] Shi L, Gong Y, Zhuo L, et al. Methyltransferase-like 3 upregulation is involved in the chemoresistance of non-small cell lung cancer. Ann Transl Med. 2022;10(3):139. 35284536 10.21037/atm-21-6608 PMC8904991

[42] Nie S, Zhang L, Liu J, et al. ALKBH5-HOXA10 loop-mediated JAK2 m6A demethylation and cisplatin resistance in epithelial ovarian cancer. J Exp Clin Cancer Res. 2021;40(1):284. 34496932 10.1186/s13046-021-02088-1 PMC8425158

[43] Chen D, Cheung H, Lau HC et al. (2022) N(6)-Methyladenosine RNA-Binding protein YTHDF1 in gastrointestinal cancers: function, molecular mechanism and clinical implication. Cancers (Basel). 14(14). 10.3390/cancers14143489 PMC9320224 35884552

[44] Cao J, Mu Q, Huang H. (2018) The Roles of Insulin-Like Growth Factor 2 mRNA-Binding Protein 2 in Cancer and Cancer Stem Cells. Stem Cells Int. 2018:4217259. 10.1155/2018/4217259 PMC5874980 29736175

[45] Betson N, Hajahmed M, Gebretsadek T, et al. Inhibition of insulin-like growth factor 2 mRNA-binding protein 1 sensitizes colorectal cancer cells to chemotherapeutics. FASEB Bioadv. 2022;4(12):816–29. 36479210 10.1096/fba.2021-00069 PMC9721091

[46] Xia C, Li Q, Cheng X, et al. Insulin-like growth factor 2 mRNA-binding protein 2-stabilized long non-coding RNA taurine up-regulated gene 1 (TUG1) promotes cisplatin-resistance of colorectal cancer via modulating autophagy. Bioengineered. 2022;13(2):2450–69. 35014946 10.1080/21655979.2021.2012918 PMC8973703

[47] Yang L, Yan B, Qu L, et al. IGF2BP3 regulates TMA7-mediated Autophagy and Cisplatin Resistance in Laryngeal Cancer via m6A RNA methylation. Int J Biol Sci. 2023;19(5):1382–400. 37056932 10.7150/ijbs.80921 PMC10086756

[48] Peres LA, da Cunha AD Jr. Acute nephrotoxicity of cisplatin: molecular mechanisms. J Bras Nefrol. 2013;35(4):332–40. 24402113 10.5935/0101-2800.20130052

[49] Wang L, Mosel AJ, Oakley GG, et al. Deficient DNA damage signaling leads to chemoresistance to cisplatin in oral cancer. Mol Cancer Ther. 2012;11(11):2401–9. 22973056 10.1158/1535-7163.MCT-12-0448 PMC3496048

[50] Zhang W, Xiao P, Tang J, et al. m6A Regulator-mediated tumour infiltration and methylation modification in Cervical Cancer Microenvironment. Front Immunol. 2022;13:888650. 35572541 10.3389/fimmu.2022.888650 PMC9098799

[51] Shi K, Chen Y, Liu R, et al. NFIC mediates m6A mRNA methylation to orchestrate transcriptional and post-transcriptional regulation to represses malignant phenotype of non-small cell lung cancer cells. Cancer Cell Int. 2024;24(1):223. 38943137 10.1186/s12935-024-03414-1 PMC11212411

[52] Lin Z, Niu Y, Wan A, et al. RNA m(6) a methylation regulates sorafenib resistance in liver cancer through FOXO3-mediated autophagy. EMBO J. 2020;39(12):e103181. 32368828 10.15252/embj.2019103181 PMC7298296

[53] Zhang F, Ran Y, Tahir M, et al. Regulation of N6-methyladenosine (m6A) RNA methylation in microglia-mediated inflammation and ischemic stroke. Front Cell Neurosci. 2022;16:955222. 35990887 10.3389/fncel.2022.955222 PMC9386152

[54] Liu L, He J, Sun G, et al. The N6-methyladenosine modification enhances ferroptosis resistance through inhibiting SLC7A11 mRNA deadenylation in hepatoblastoma. Clin Transl Med. 2022;12(5):e778. 35522946 10.1002/ctm2.778 PMC9076012

[55] Murakami S, Jaffrey SR. Hidden codes in mRNA: control of gene expression by m(6)a. Mol Cell. 2022;82(12):2236–51. 35714585 10.1016/j.molcel.2022.05.029 PMC9216239

[56] Tian S, Lai J, Yu T, et al. Regulation of Gene expression Associated with the N6-Methyladenosine (m6A) enzyme system and its significance in Cancer. Front Oncol. 2020;10:623634. 33552994 10.3389/fonc.2020.623634 PMC7859513

[57] Kang HG, Kim WJ, Noh MG et al. (2020) SPON2 is upregulated through Notch Signaling Pathway and promotes Tumor Progression in Gastric Cancer. Cancers (Basel). 12(6). 10.3390/cancers12061439 PMC7352369 32492954

[58] Wu M, Kong D, Zhang Y. SPON2 promotes the bone metastasis of lung adenocarcinoma via activation of the NF-kappaB signaling pathway. Bone. 2023;167:116630. 36427776 10.1016/j.bone.2022.116630

[59] Huang C, Ou R, Chen X, et al. Tumor cell-derived SPON2 promotes M2-polarized tumor-associated macrophage infiltration and cancer progression by activating PYK2 in CRC. J Exp Clin Cancer Res. 2021;40(1):304. 34583750 10.1186/s13046-021-02108-0 PMC8477524

[60] Li H, Luo F, Jiang X et al. (2022) CircITGB6 promotes ovarian cancer cisplatin resistance by resetting tumor-associated macrophage polarization toward the M2 phenotype. J Immunother Cancer 10(3). 10.1136/jitc-2021-004029 PMC8919471 35277458

[61] Prenen H, Mazzone M. Tumor-associated macrophages: a short compendium. Cell Mol Life Sci. 2019;76(8):1447–58. 30747250 10.1007/s00018-018-2997-3 PMC11105658

