# Supplementary figures and images for: Knockdown of IGF2BP2 overcomes cisplatin-resistance in lung cancer through downregulating Spon2 gene
Source: Hereditas. 2024 Dec 28;161:55. doi: 10.1186/s41065-024-00360-w (PMC11681704; doi:10.1186/s41065-024-00360-w)

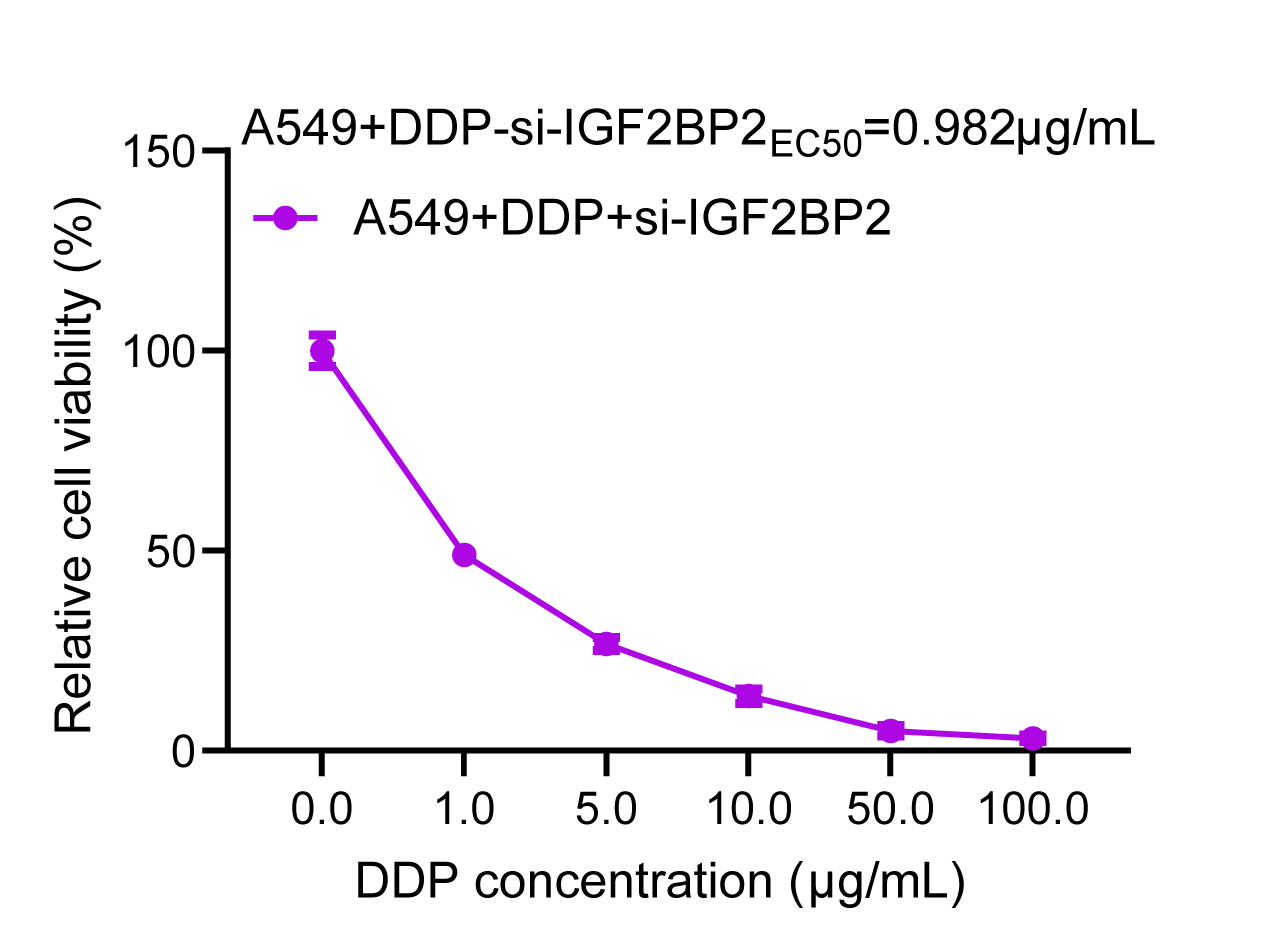

Supplement: Supplementary file 1 — Supplementary Material 1: Figure S1: Knockdown of IGF2BP2 augmented DDP cytotoxicity on A549 cells. si-IGF2BP2-transfected A549 cells were treated with DDP (0, 1, 5, 10, 50, or 100 μg/mL) for 48 h. Cell viability was assessed using the MTT assay. [file 41065_2024_360_MOESM1_ESM.tif]

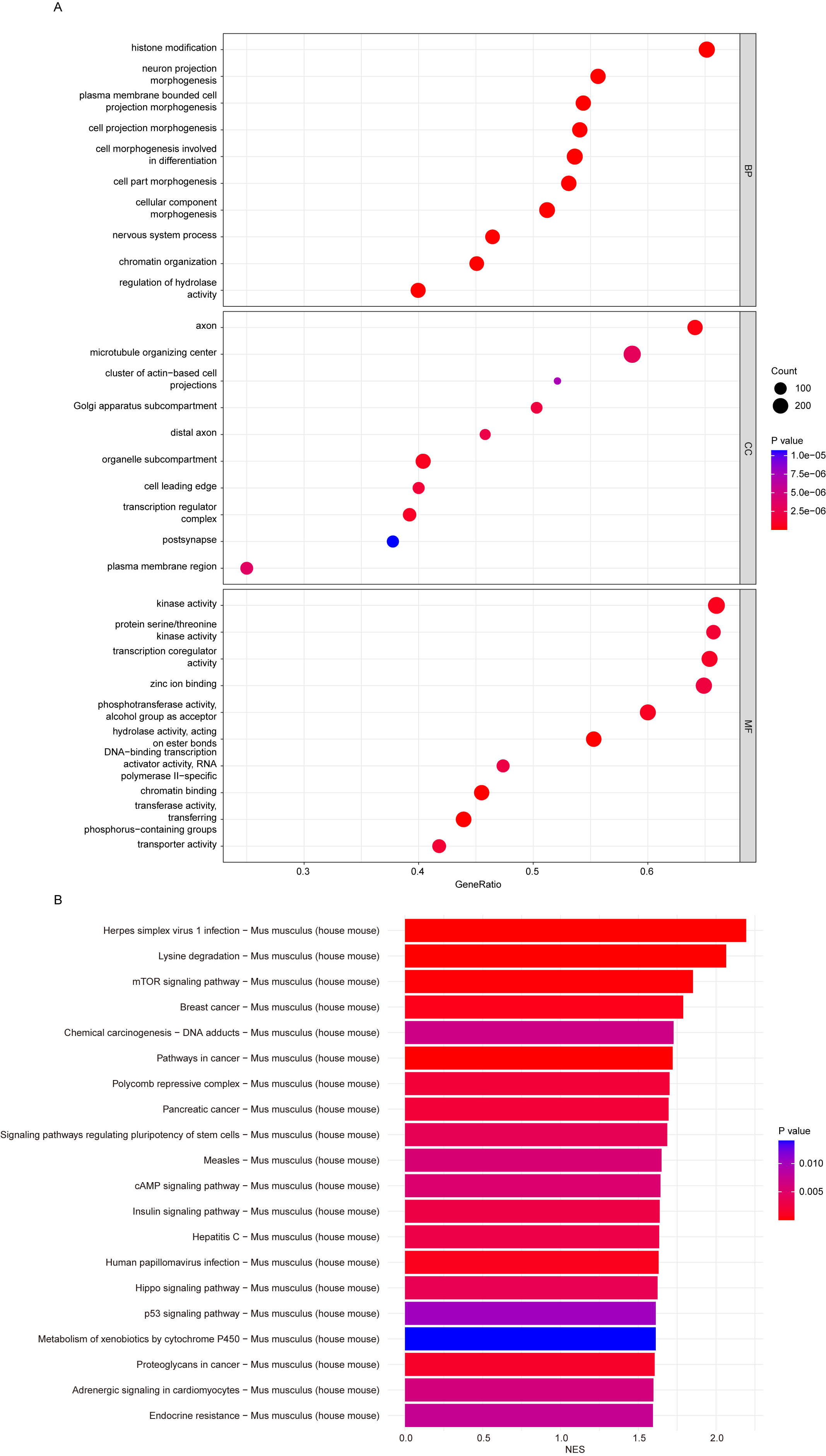

Supplement: Supplementary file 2 — Supplementary Material 2 Figure S2: Functional enrichment analyses. (A) GSEA-GO and (B) GSEA-KEGG enrichment analysis. [file 41065_2024_360_MOESM2_ESM.tif]
