# Supplementary material for: Knockdown of IGF2BP2 overcomes cisplatin-resistance in lung cancer through downregulating Spon2 gene
Source: Hereditas. 2024 Dec 28;161:55. doi: 10.1186/s41065-024-00360-w (PMC11681704; doi:10.1186/s41065-024-00360-w)

Figure 1B
IGF2BP2

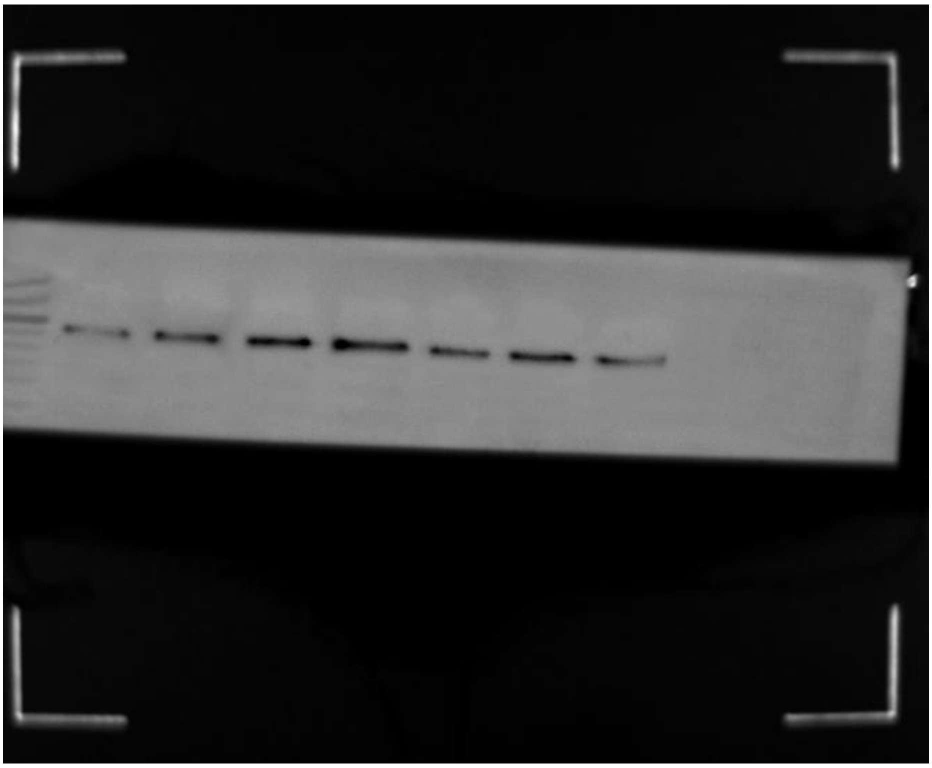

β-actin

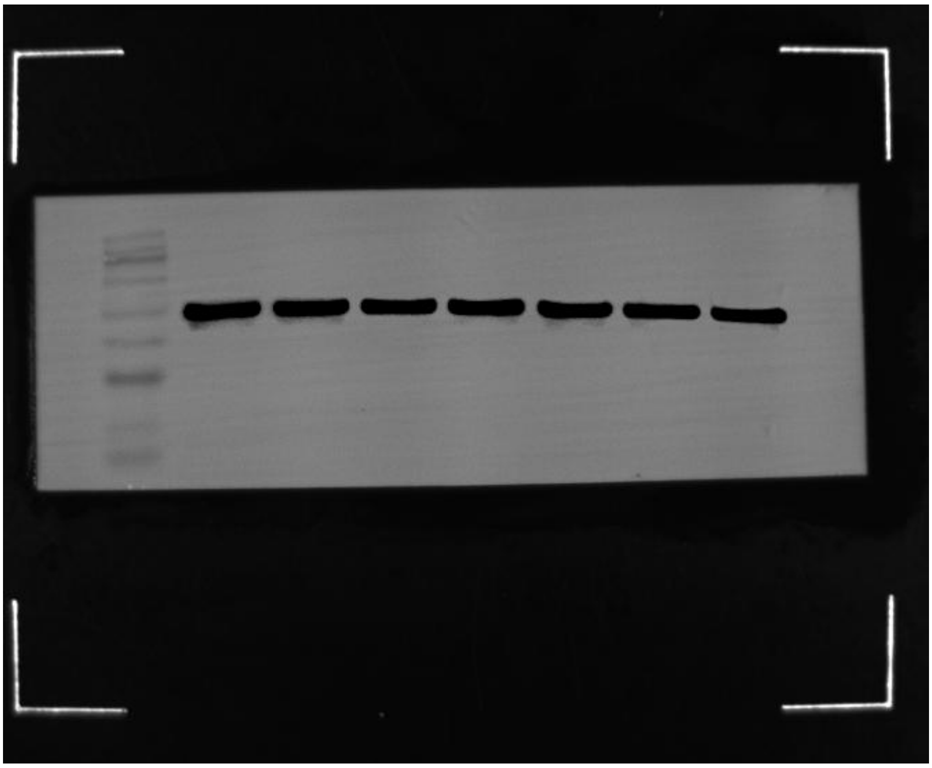

Figure 1D

IGF2BP2

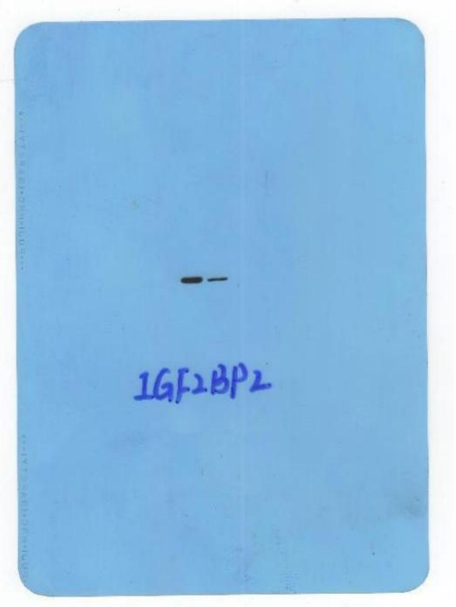

GAPDH

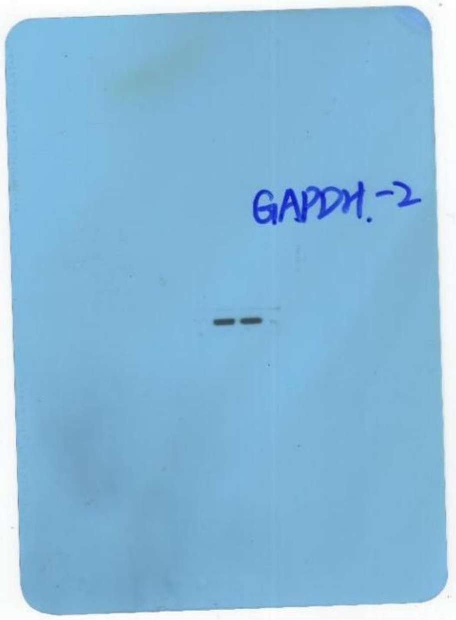

Figure 8A
Spon2

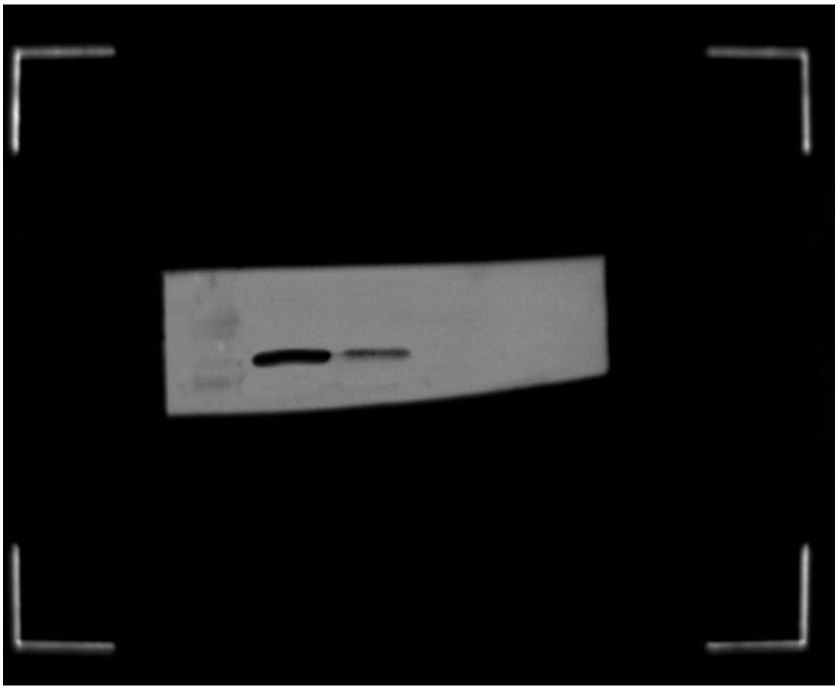

GAPDH

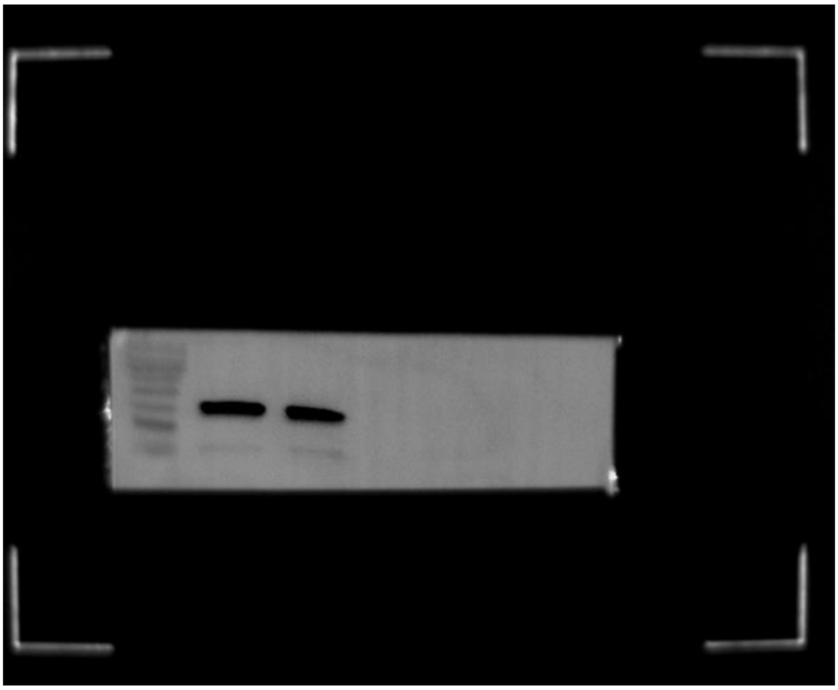

Figure 8B
Spon2

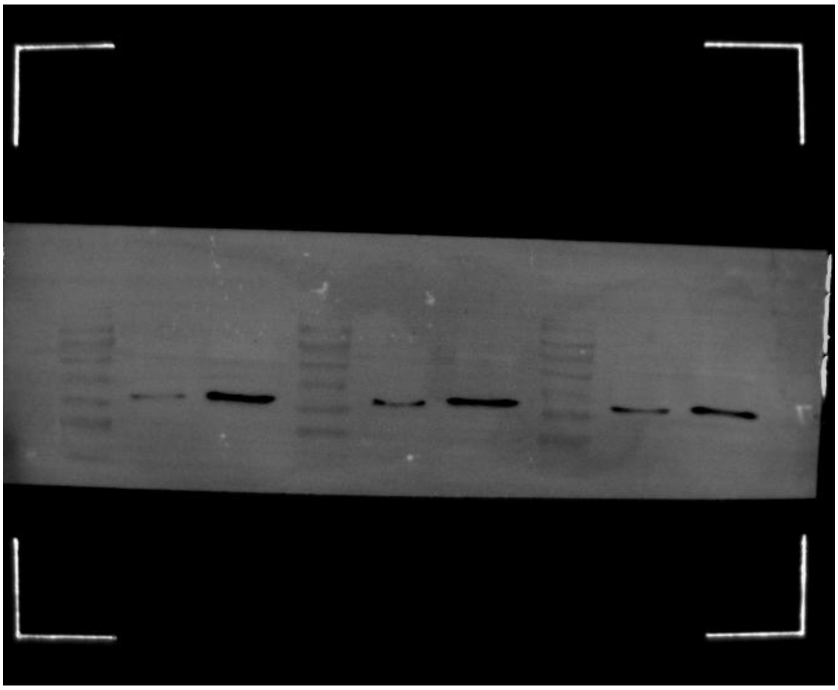

GAPDH

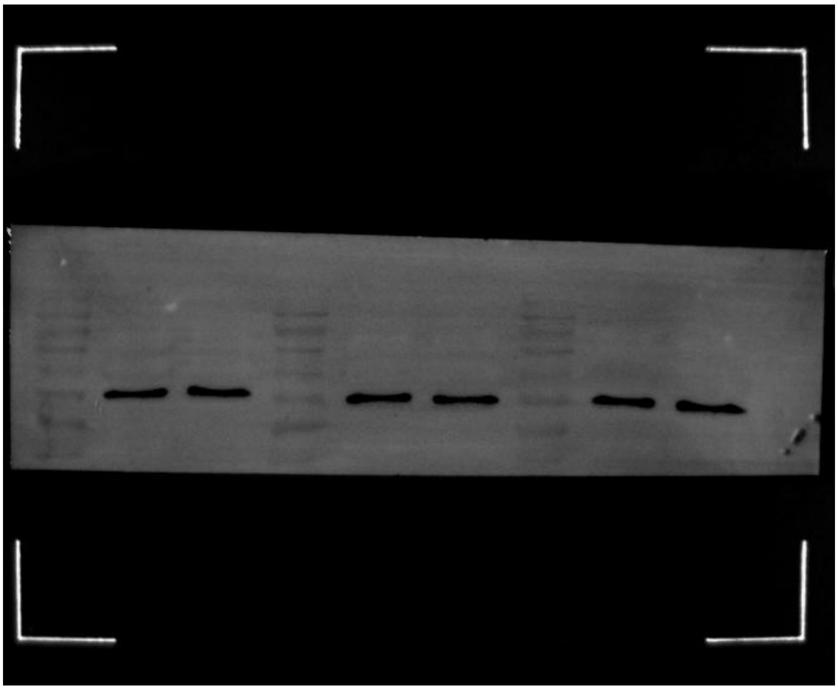

Figure 8C
Spon2

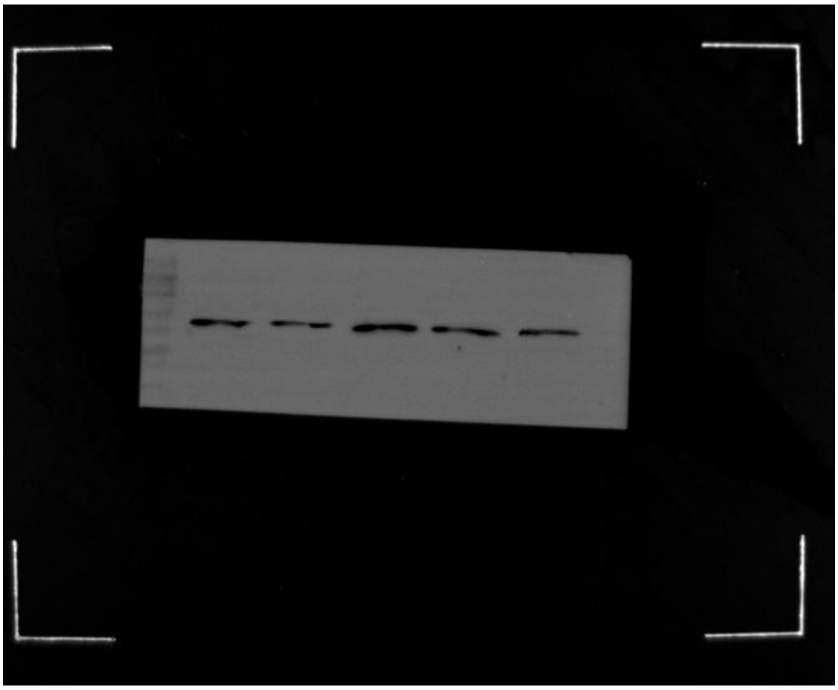

GAPDH

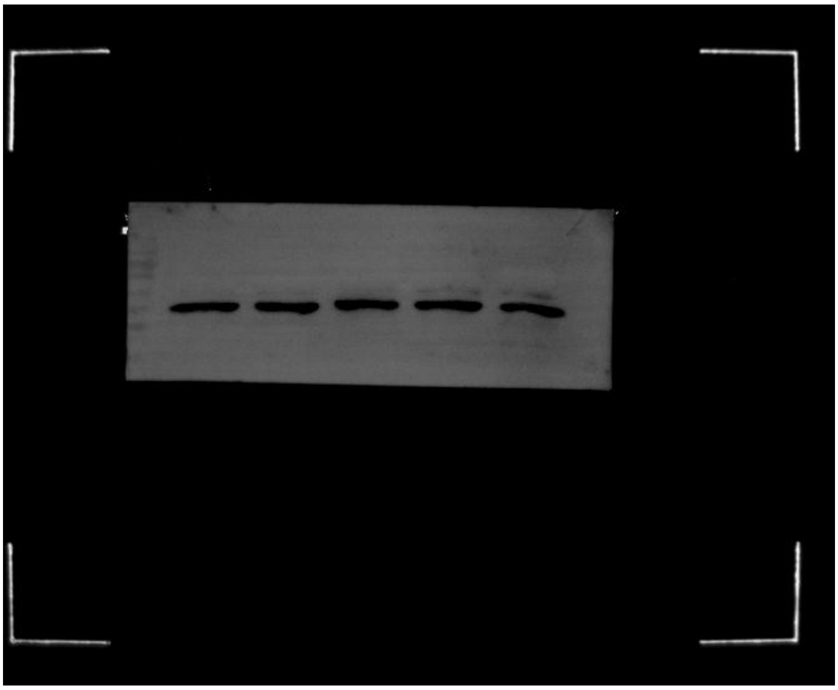

Figure 8D
Spon2

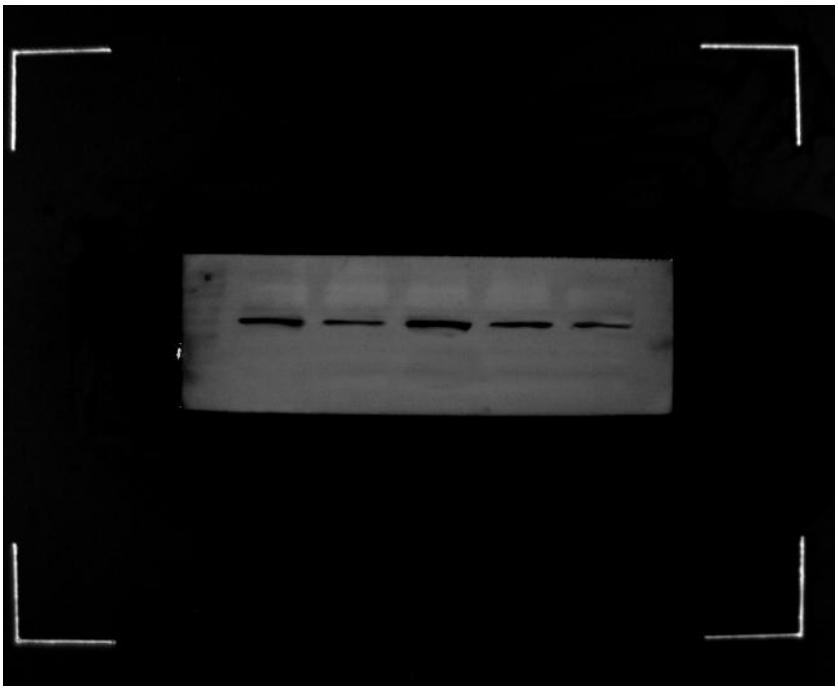

GAPDH

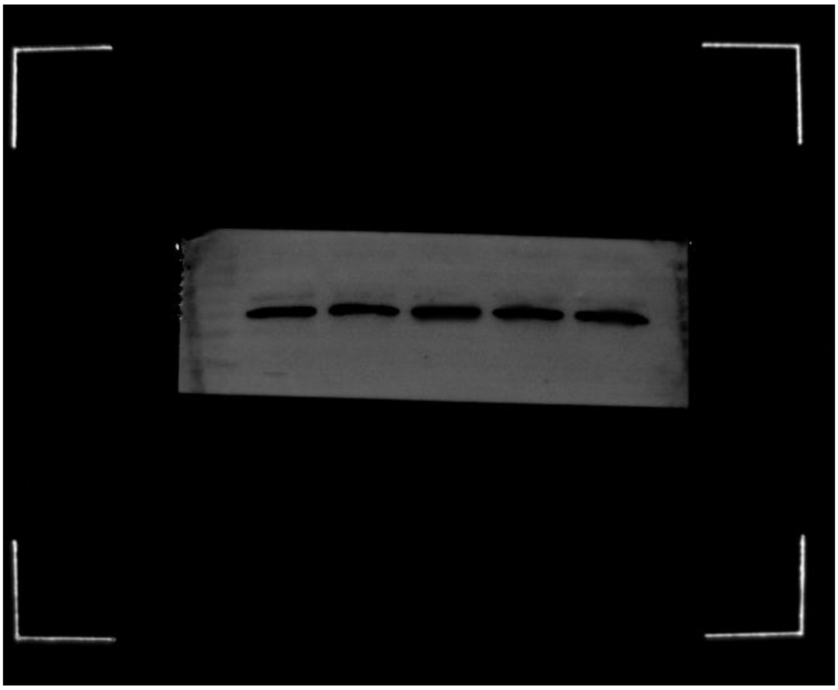

Supplement: Supplementary file 3 — Supplementary Material 3 [file 41065_2024_360_MOESM3_ESM.pdf]
