# Supplementary material for: Knockdown of IGF2BP2 overcomes cisplatin-resistance in lung cancer through downregulating Spon2 gene
Source: Hereditas. 2024 Dec 28;161:55. doi: 10.1186/s41065-024-00360-w (PMC11681704; doi:10.1186/s41065-024-00360-w)
Supplement: Supplementary file 4 — Supplementary Material 4: Table S1: The sequences of si-IGF2BP2, oe-IGF2BP2, oe-Spon2. [file 41065_2024_360_MOESM4_ESM.docx]

**Table S1** The sequences of si-IGF2BP2, oe-IGF2BP2, oe-Spon2

| si-IGF2BP2 | 5′-ACAGGACUGUCCGUGCUAUTT-3′ |
| --- | --- |
| oe-IGF2BP2 | atgatgaa caagctttac atcgggaacc tgagccccgc  121 cgtcaccgcc gacgacctcc ggcagctctt tggggacagg aagctgcccc tggcgggaca  181 ggtcctgctg aagtccggct acgccttcgt ggactacccc gaccagaact gggccatccg  241 cgccatcgag accctctcgg gtaaagtgga attgcatggg aaaatcatgg aagttgatta  301 ctcagtctct aaaaagctaa ggagcaggaa aattcagatt cgaaacatcc ctcctcacct  361 gcagtgggag gtgttggatg gacttttggc tcaatatggg acagtggaga atgtggaaca  421 agtcaacaca gacacagaaa ccgccgttgt caacgtcaca tatgcaacaa gagaagaagc  481 aaaaatagcc atggagaagc taagcgggca tcagtttgag aactactcct tcaagatttc  541 ctacatcccg gatgaagagg tgagctcccc ttcgccccct cagcgagccc agcgtgggga  601 ccactcttcc cgggagcaag gccacgcccc tgggggcact tctcaggcca gacagattga  661 tttcccgctg cggatcctgg tccccaccca gtttgttggt gccatcatcg gaaaggaggg  721 cttgaccata aagaacatca ctaagcagac ccagtcccgg gtagatatcc atagaaaaga  781 gaactctgga gctgcagaga agcctgtcac catccatgcc accccagagg ggacttctga  841 agcatgccgc atgattcttg aaatcatgca gaaagaggca gatgagacca aactagccga  901 agagattcct ctgaaaatct tggcacacaa tggcttggtt ggaagactga ttggaaaaga  961 aggcagaaat ttgaagaaaa ttgaacatga aacagggacc aagataacaa tctcatcttt  1021 gcaggatttg agcatataca acccggaaag aaccatcact gtgaagggca cagttgaggc  1081 ctgtgccagt gctgagatag agattatgaa gaagctgcgt gaggcctttg aaaatgatat  1141 gctggctgtt aacacccact ccggatactt ctccagcctg tacccccatc accagtttgg  1201 cccgttcccg catcatcact cttatccaga gcaggagatt gtgaatctct tcatcccaac  1261 ccaggctgtg ggcgccatca tcgggaagaa gggggcacac atcaaacagc tggcgagatt  1321 cgccggagcc tctatcaaga ttgcccctgc ggaaggccca gacgtcagcg aaaggatggt  1381 catcatcacc gggccaccgg aagcccagtt caaggcccag ggacggatct ttgggaaact  1441 gaaagaggaa aacttcttta accccaaaga agaagtgaag ctggaagcgc atatcagagt  1501 gccctcttcc acagctggcc gggtgattgg caaaggtggc aagaccgtga acgaactgca  1561 gaacttaacc agtgcagaag tcatcgtgcc tcgtgaccaa acgccagatg aaaatgagga  1621 agtgatcgtc agaattatcg ggcacttctt tgctagccag actgcacagc gcaagatcag  1681 ggaaattgta caacaggtga agcagcagga gcagaaatac cctcagggag tcgcctcaca  1741 gcgcagcaag tga |
| oe-Spon2 | atggaaaacc ccagcccggc cgccgccctg ggcaaggccc tctgcgctct cctcctggcc  301 actctcggcg ccgccggcca gcctcttggg ggagagtcca tctgttccgc cagagccctg  361 gccaaataca gcatcacctt cacgggcaag tggagccaga cggccttccc caagcagtac  421 cccctgttcc gcccccctgc gcagtggtct tcgctgctgg gggccgcgca tagctccgac  481 tacagcatgt ggaggaagaa ccagtacgtc agtaacgggc tgcgcgactt tgcggagcgc  541 ggcgaggcct gggcgctgat gaaggagatc gaggcggcgg gggaggcgct gcagagcgtg  601 cacgcggtgt tttcggcgcc cgccgtcccc agcggcaccg ggcagacgtc ggcggagctg  661 gaggtgcagc gcaggcactc gctggtctcg tttgtggtgc gcatcgtgcc cagccccgac  721 tggttcgtgg gcgtggacag cctggacctg tgcgacgggg accgttggcg ggaacaggcg  781 gcgctggacc tgtaccccta cgacgccggg acggacagcg gcttcacctt ctcctccccc  841 aacttcgcca ccatcccgca ggacacggtg accgagataa cgtcctcctc tcccagccac  901 ccggccaact ccttctacta cccgcggctg aaggccctgc ctcccatcgc cagggtgaca  961 ctggtgcggc tgcgacagag ccccagggcc ttcatccctc ccgccccagt cctgcccagc  1021 agggacaatg agattgtaga cagcgcctca gttccagaaa cgccgctgga ctgcgaggtc  1081 tccctgtggt cgtcctgggg actgtgcgga ggccactgtg ggaggctcgg gaccaagagc  1141 aggactcgct acgtccgggt ccagcccgcc aacaacggga gcccctgccc cgagctcgaa  1201 gaagaggctg agtgcgtccc tgataactgc gtctaa |
